# Effectiveness and cost-effectiveness against malaria of three types of dual-active-ingredient long-lasting insecticidal nets (LLINs) compared with pyrethroid-only LLINs in Tanzania: a four-arm, cluster-randomised trial

**DOI:** 10.1016/S0140-6736(21)02499-5

**Published:** 2022-03-26

**Authors:** Jacklin F Mosha, Manisha A Kulkarni, Eliud Lukole, Nancy S Matowo, Catherine Pitt, Louisa A Messenger, Elizabeth Mallya, Mohamed Jumanne, Tatu Aziz, Robert Kaaya, Boniface A Shirima, Gladness Isaya, Monica Taljaard, Jacklin Martin, Ramadhan Hashim, Charles Thickstun, Alphaxard Manjurano, Immo Kleinschmidt, Franklin W Mosha, Mark Rowland, Natacha Protopopoff

**Affiliations:** a Department of Parasitology, National Institute for Medical Research, Mwanza Medical Research Centre, Mwanza, Tanzania; b School of Epidemiology and Public Health, University of Ottawa, Ottawa, ON, Canada; c Department of Disease Control, London School of Hygiene & Tropical Medicine, London, UK; d Department of Global Health and Development, London School of Hygiene & Tropical Medicine, London, UK; e MRC International Statistics and Epidemiology Group, London School of Hygiene & Tropical Medicine, London, UK; f Department of Parasitology, Kilimanjaro Christian Medical University College, Moshi, Tanzania; g Ottawa Hospital Research Institute, Ottawa, ON, Canada; h Wits Research Institute for Malaria, School of Pathology, Faculty of Health Sciences, University of the Witwatersrand, Johannesburg, South Africa; i Southern African Development Community Malaria Elimination Eight Secretariat, Windhoek, Namibia

## Abstract

**Background:**

Long-lasting insecticidal nets (LLINs) have successfully reduced malaria in sub-Saharan Africa, but their effectiveness is now partly compromised by widespread resistance to insecticides among vectors. We evaluated new classes of LLINs with two active ingredients with differing modes of action against resistant malaria vectors.

**Methods:**

We did a four-arm, cluster-randomised trial in Misungwi, Tanzania. Clusters were villages, or groups of hamlets, with at least 119 households containing children aged 6 months to 14 years living in the cluster's core area. Constrained randomisation was used to allocate clusters (1:1:1:1) to receive one of four types of LLIN treated with the following: α-cypermethrin only (pyrethroid-only [reference] group); pyriproxyfen and α-cypermethrin (pyriproxyfen group); chlorfenapyr and α-cypermethrin (chlorfenapyr group); or the synergist piperonyl butoxide and permethrin (piperonyl butoxide group). At least one LLIN was distributed for every two people. Community members and the field team were masked to group allocation. Malaria prevalence data were collected through cross-sectional surveys of randomly selected households from each cluster, in which children aged 6 months to 14 years were assessed for *Plasmodium falciparum* malaria infection by rapid diagnostic tests. The primary outcome was malaria infection prevalence at 24 months after LLIN distribution, comparing each of the dual-active-ingredient LLINs to the standard pyrethroid-only LLINs in the intention-to-treat population. The primary economic outcome was cost-effectiveness of dual-active-ingredient LLINs, based on incremental cost per disability-adjusted life-year (DALY) averted compared with pyrethroid-only LLINs, modelled over a 2-year period; we included costs of net procurement and malaria diagnosis and treatment, and estimated DALYs in all age groups. This study is registered with ClinicalTrials.gov (NCT03554616), and is ongoing but no longer recruiting.

**Findings:**

84 clusters comprising 39 307 households were included in the study between May 11 and July 2, 2018. 147 230 LLINs were distributed among households between Jan 26 and Jan 28, 2019. Use of study LLINs was reported in 3155 (72·1%) of 4378 participants surveyed at 3 months post-distribution and decreased to 8694 (40·9%) of 21 246 at 24 months, with varying rates of decline between groups. Malaria infection prevalence at 24 months was 549 (45·8%) of 1199 children in the pyrethroid-only reference group, 472 (37·5%) of 1258 in the pyriproxyfen group (adjusted odds ratio 0·79 [95% CI 0·54–1·17], p=0·2354), 512 (40·7%) of 1259 in the piperonyl butoxide group (0·99 [0·67–1·45], p=0·9607), and 326 [25·6%] of 1272 in the chlorfenapyr group (0·45 [0·30–0·67], p=0·0001). Skin irritation or paraesthesia was the most commonly reported side-effect in all groups. Chlorfenapyr LLINs were the most cost-effective LLINs, costing only US$19 (95% uncertainty interval 1–105) more to public providers or $28 (11–120) more to donors per DALY averted over a 2-year period compared with pyrethroid-only LLINs, and saving costs from societal and household perspectives.

**Interpretation:**

After 2 years, chlorfenapyr LLINs provided significantly better protection than pyrethroid-only LLINs against malaria in an area with pyrethroid-resistant mosquitoes, and the additional cost of these nets would be considerably below plausible cost-effectiveness thresholds ($292–393 per DALY averted). Before scale-up of chlorfenapyr LLINs, resistance management strategies are needed to preserve their effectiveness. Poor textile and active ingredient durability in the piperonyl butoxide and pyriproxyfen LLINs might have contributed to their relative lack of effectiveness compared with standard LLINs.

**Funding:**

Joint Global Health Trials scheme (UK Foreign, Commonwealth and Development Office; UK Medical Research Council; Wellcome; UK Department of Health and Social Care), US Agency for International Development, President's Malaria Initiative.

**Research in context**

**Evidence before this study**

We searched PubMed on May 12, 2021, with no language or date restrictions. We used the search term “randomised controlled trial” and “malaria” and “net” or “long lasting insecticidal net”, combined with the terms “piperonyl butoxide” or “pyriproxyfen” or “chlorfenapyr”. We identified two cluster-randomised controlled trials that evaluated the efficacy with regard to malaria outcomes of long-lasting insecticidal nets (LLINs) combining the synergist piperonyl butoxide and a pyrethroid insecticide (one conducted in Tanzania and one in Uganda), and a meta-analysis of those results in a Cochrane review. One randomised controlled trial was found assessing the effectiveness of LLINs treated with pyriproxyfen and pyrethroid, in Burkina Faso, but we found no trials of LLINs combining a pyrethroid and chlorfenapyr. The Tanzanian study was a four-arm superiority trial comparing piperonyl butoxide LLINs (Olyset Plus) with a standard pyrethroid-only LLIN (Olyset net), with or without indoor residual spraying, using a two-by-two factorial design. The Ugandan trial compared piperonyl butoxide LLINs of two types, Olyset Plus (permethrin and piperonyl butoxide) and Permanet 3.0 (deltamethrin, and piperonyl butoxide only on the roof panel of the net) with standard LLINs of the same brands but without the piperonyl butoxide. Both trials showed that piperonyl butoxide LLINs reduced malaria prevalence in children compared with the standard LLIN, up to 18 months post-intervention in Uganda and 21 months in Tanzania. The Cochrane review concluded that, for these two trials, the odds of malaria infection was 31% lower in the piperonyl butoxide LLIN groups than in the standard LLIN groups at 21–25 months. Intensity of insecticide resistance in malaria vectors was high in both study areas. The trial in Burkina Faso used a stepped-wedge design and showed a small (12%) but significant reduction in clinical malaria incidence in children in an area with pyriproxyfen LLINs compared with those residing in the standard pyrethroid-only LLIN group, whereas there was no effect of the pyriproxyfen LLINs on malaria prevalence.

**Added value of this study**

To our knowledge, this study is the first to assess the effectiveness and cost-effectiveness of the three classes of dual-active-ingredient LLINs currently available for malaria control. The use of a mixture of active ingredients on an LLIN has the potential to be more effective in controlling malaria transmitted by insecticide-resistant vectors. This strategy can, in turn, delay the evolution of resistance, and therefore extend the effective lifespan of both active ingredients. We showed for the first time, since the adoption of pyrethroid insecticides for malaria control 40 years ago, that another insecticide, chlorfenapyr, when used for net treatment, is a safe, effective, and cost-effective alternative to standard pyrethroid-only LLINs. Pyrethroid LLINs treated with pyriproxyfen, an insect growth regulator designed to sterilise female mosquitoes, did not provide additional protection against malaria compared with standard pyrethroid-only LLINs. Moderate use of this LLIN in the trial, low residual bioefficacy of pyriproxyfen on the surface of the net fabric, or pyriproxyfen resistance in malaria vectors, or a combination of these factors, might explain this result. Finally, our trial confirmed the superior effectiveness of piperonyl butoxide LLINs compared with standard pyrethroid-only LLINs, but over a limited period of 12 months, possibly due to low textile durability leading to rapid reduction in the use of this net in the community.

**Implications of all the available evidence**

This study has several major implications. First, LLINs treated with both chlorfenapyr and a pyrethroid should be deployed in areas of insecticide resistance, with careful consideration of strategies for resistance management. Second, further investigation of the role of cross-resistance in pyriproxyfen LLINs is warranted to understand the utility of these nets for malaria control in different ecological and epidemiological settings. Finally, funders, national malaria control programmes, and stakeholders should urgently advocate and set up incentive structures to promote better textile and active ingredient durability on LLINs to maximise the potential of these tools for sustained malaria control in the face of rising insecticide resistance.

## Introduction

Long-lasting insecticidal nets (LLINs) have successfully controlled malaria in sub-Saharan Africa for two decades, but their effectiveness might now be compromised by widespread resistance to pyrethroid insecticides in Anopheles vectors.^1^ Until 2017, pyrethroids were the only insecticide class recommended by WHO for use in LLINs because of their safety profile. New insecticides are now needed to sustain effective malaria vector control. To receive a WHO public health recommendation, new LLIN classes need to demonstrate effectiveness superior to that of standard LLINs against malaria indicators in two randomised controlled trials (RCTs).^2^ In 2017, WHO recommended the first new class of dual-active-ingredient LLINs, treated with a mixture of pyrethroid insecticide and the synergist piperonyl butoxide, which enhances the killing property of the insecticide.^3^ In RCTs, the piperonyl butoxide nets reduced malaria prevalence by 44% in Tanzania^4^ and 27% in Uganda^5^ compared to standard pyrethroid LLINs in areas of pyrethroid resistance. Piperonyl butoxide nets have now started to be deployed in national campaigns across sub-Saharan Africa.^6^

LLINs combining a pyrethroid and a second insecticide with a different mode of action have also been developed. This class of LLINs might be more effective than standard pyrethroid-only LLINs in controlling pyrethroid-resistant malaria vectors, and have the potential to delay the evolution of resistance, thereby extending the effective lifespan of this class of product.^7^ One class combines a pyrethroid with pyriproxyfen, an insect growth regulator that sterilises adult female mosquitoes. New and unwashed pyriproxyfen nets have shown up to 83% sterilisation of malaria vectors in Benin.^8^, ^9^ In an RCT in Burkina Faso, pyriproxyfen nets had significantly greater impact on the clinical incidence of malaria than did pyrethroid-only LLINs.^10^ Another class of dual-active-ingredient LLIN that mixes two insecticides, a pyrethroid and the pyrrole chlorfenapyr, enhanced the killing of resistant vectors compared with pyrethroid-only LLINs in experimental hut studies,^11^, ^12^ but has not previously been evaluated for its public health impact.

Our trial compared the effectiveness of these three classes of dual-active-ingredient LLINs to pyrethroid-only LLINs in an area of Tanzania with pyrethroid-resistant mosquitoes. We assessed malaria infection prevalence through repeated cross-sectional surveys. We also evaluated clinical malaria incidence using a nested cohort study, malaria transmission through entomological monitoring, and cost-effectiveness by modelling from incidence estimates.

## Methods

### Study design and participants

We did a four parallel-arm, superiority, cluster-randomised, controlled trial in the Misungwi district of Mwanza, northwest Tanzania. Previous vector control interventions in the region included indoor residual spraying and a universal LLIN coverage campaign in 2015, as well as distribution of LLINs to primary school students in 2018 and to pregnant women during antenatal care visits.

Clusters were allocated to one of four study groups: the pyrethroid-only (reference) group, which received LLINs containing the pyrethroid α-cypermethrin (5 g/kg; Interceptor, BASF SE, Ludwigshafen Germany); the pyriproxyfen group, with LLINs combining pyriproxyfen (5·5 g/kg) and α-cypermethrin (5·5 g/kg; Royal Guard, Disease Control Technologies, Greer, SC, USA); the chlorfenapyr group, with LLINs combining chlorfenapyr (4·8 g/kg) and α-cypermethrin (2·4 g/kg; Interceptor G2, BASF SE, Ludwigshafen, Germany); and the piperonyl butoxide group, with LLINs combining piperonyl butoxide (10 g/kg) and the pyrethroid permethrin (20 g/kg; Olyset Plus, Sumitomo Chemical, Tokyo, Japan).

A cluster was defined in the protocol as a village or group of hamlets with a minimum of 150 households containing children aged 6 months to 14 years living in the core area; this number was later reduced to 119 households during mapping and census. Clusters were designed with core and buffer areas, with all houses in core areas located at least 600 m from any houses in adjacent clusters to reduce contamination of intervention effects between study groups. Houses allocated to cluster buffer areas received the same LLINs as those distributed in the core area, but outcome monitoring was done only in households in the core areas. Each household in the study area was enumerated and mapped using GPS handheld units and a short Open Data Kit programmed questionnaire.^13^

**Figure 1:**
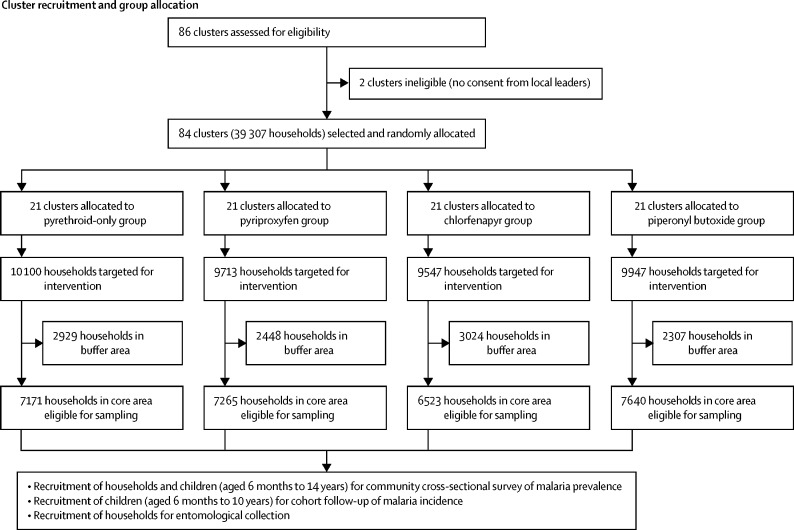

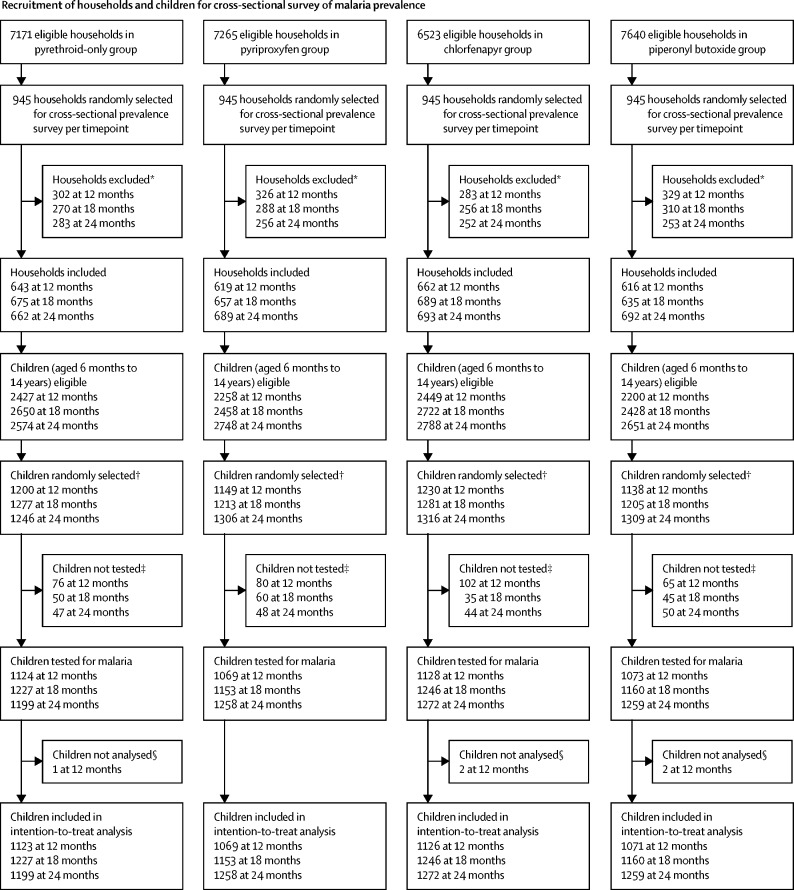

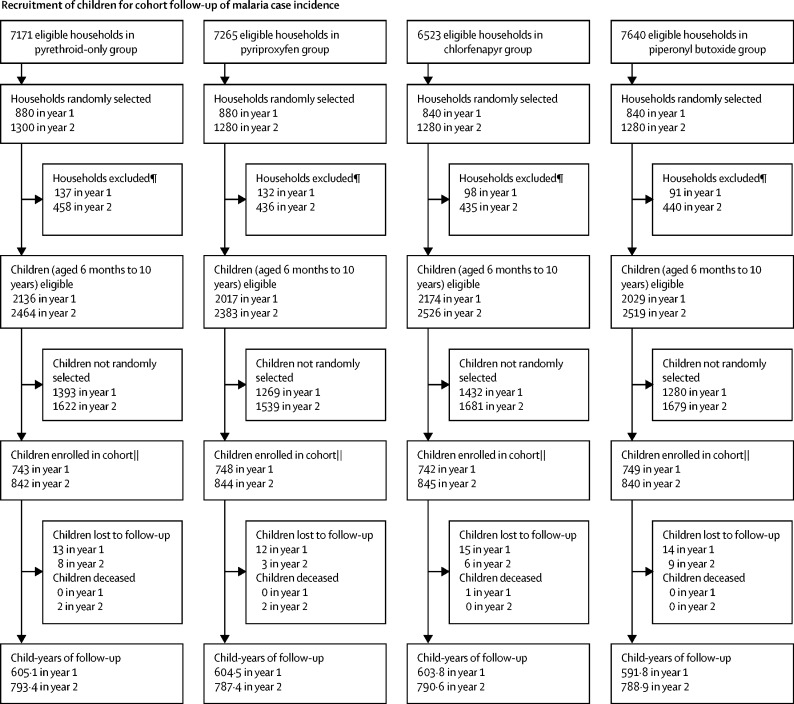

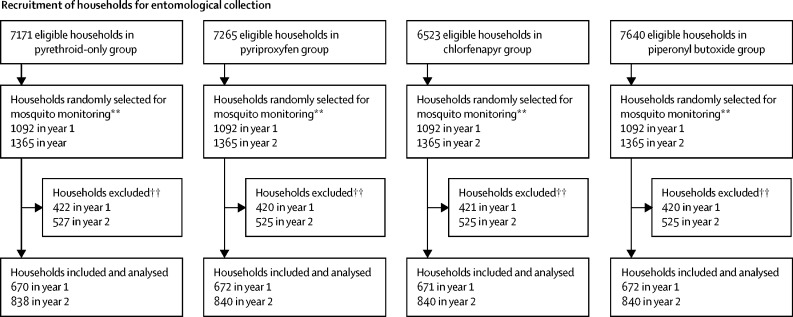
Trial profile *See appendix (p 11) for a breakdown of reasons for exclusion. † From each household, up to two children were selected for rapid diagnostic testing. ‡Children did not attend clinical appointment and were not tested for malaria. §Malaria test results missing. ¶Exclusion criteria were the same as for cross-sectional prevalence surveys; however, as community health workers only recorded the consenting households, a breakdown of reasons for exclusion is unavailable. ||Some community health workers enrolled more than 35 or 40 households per cluster per year. **13 households per cluster were selected at each round of collections (four rounds in year 1 and five rounds in year 2); a fifth round of collections was added in year 2 to compensate for the suspension of collections between March and May, 2020, due to the SARS-CoV-2 pandemic. †† See appendix (p 18) for a breakdown of reasons for exclusion.

We did cross-sectional surveys to collect data on household factors and malaria infection prevalence at baseline (October 2018) and at 12 months (January, 2020), 18 months (August, 2020), and 24 months (January, 2021) after LLIN distribution (figure 1). At each timepoint, 45 households were randomly selected from the census listing of each cluster's core area using swor command in Stata (version 15). In each household, up to two children aged 6 months to 14 years were randomly selected for detection of malaria parasitaemia using random number tables.

To assess malaria case incidence, 35 households per cluster were enrolled after LLIN distribution in year 1. 40 households were selected at random, and these households were visited in order of the randomisation until we obtained 35 consenting households, at which point enrolment stopped. Five additional households were selected if needed to reach 35 consenting households. From each household, one child aged 6 months to 10 years was selected at random and actively followed up for 1 year. A second independent cohort of 40 children per cluster (number increased because of the low incidence in the first year) was recruited 1 year after distribution and also followed up for 1 year. A similar recruitment process was followed, with 40 households initially selected, and an additional 20 selected at random until 40 consented.

Routine mosquito collections were conducted in eight randomly selected households in 28 clusters per month (seven clusters per intervention group) for one night. Each collection round in the 84 clusters lasted for 3 months, with successive rounds over a 2-year period; five additional houses were also randomly selected but only visited if any of the eight selected households were ineligible or unavailable for inclusion. From the five additional households, the one the closest (by distance, according to GPS) to the ineligible household from the original eight selected was visited.

Inclusion criteria for prevalence and incidence data collection were households with at least one child of appropriate age who permanently resided in the selected household and an adult caregiver who could provide written consent. All households in the core area were eligible for mosquito collection. Exclusion criteria were dwellings not found or vacant, no adult caregiver capable of giving informed consent, or eligible children who were severely ill.

Institutional review boards of the Tanzanian National Institute for Medical Research (reference NIMR/HQ/R.8a/Vol.IX/2743), Kilimanjaro Christian Medical University College (2267), London School of Hygiene & Tropical Medicine (14952), and University of Ottawa (H-05-19-4411) granted ethical approval. During the household census, leaders of each hamlet were informed about the distribution and asked to consent on behalf of the community. Household members could decide whether to receive a study LLIN. Written informed consent was obtained from an adult guardian for selected children and from any adult living in households selected for mosquito collection.

The protocol for this study has been published previously.^13^

### Randomisation and masking

An independent statistician conducted constrained randomisation to allocate the 84 clusters to the four study groups at a ratio of 1:1:1:1, ensuring that absolute differences in cluster means between study groups were within the specified ranges for each of the following variables: population size (range ± 9000 people); malaria infection prevalence at baseline (± 5 percentage points); socioeconomic status, measured as the percentage of households in the poorest tercile based on the wealth index of the entire study area (± 10 percentage points); LLIN use, measured as the percentage of residents who reported using a net last night (± 10 percentage points); and cluster suitability (predicted for each cluster using an ecological niche model and based on observed species composition in the study area)^13^ for *Anopheles gambiae* sensu stricto (ss) and *Anopheles funestus* (± 15 percentage points). Using Stata (version 11), 200 000 random allocations were generated and tested against the restriction criteria; of the 10 077 acceptable allocations, one was selected at random. The validity of the randomisations was verified by checking the frequency of allocations to the same study group of all pairs of clusters.

The inhabitants of each cluster and the field staff who were responsible for enrolment and collected data were masked to the type of LLIN allocated. LLINs of each type were similar in appearance apart from a colour-coded loop and a unique identifying code.

### Procedures

All households enumerated in the core and buffer areas of clusters were allocated at least one LLIN for every two people.^14^ Behaviour change communication activities were done during and after the net distribution to increase LLIN use. A survey was done in eight randomly selected houses in each cluster 3 months after distribution to assess LLIN coverage.

During cross-sectional household surveys, information on household characteristics, LLIN use, and recent malaria cases were collected using electronic forms in tablets with Open Data Kit. At 12 and 24 months, net textile integrity was assessed by counting holes in a subsample of households (13 of 45 households per cluster). All selected children who came for the clinical appointment were assessed for *Plasmodium falciparum* malaria infection using rapid diagnostic tests (CareStart malaria HRP2 [pf], DiaSys, Wokingham, UK) and for haemoglobin concentration (HemoCue Hb 201+, Aktiebolaget Leo Diagnostics, Helsigborg, Sweden). Children who tested positive for malaria were treated with artemether–lumefantrine. Any child presenting with minor illness, such as helminth infection, upper respiratory tract infection, mild anaemia, or diarrhoea, was treated. Children with severe symptoms were referred to the nearest health facility. Cross-sectional prevalence surveys were not affected by the SARS-CoV-2 pandemic.

All cohort children were treated with artemether–lumefantrine following recruitment. After 2 weeks, clearance of malaria infection was verified by rapid diagnostic tests and blood slides. Cohort children were asked to visit, with their guardian, a central point in each cluster every 2 weeks, or monthly during the dry season (July–August). Children with fever (tympanic temperature ≥37·5°C) or a history of fever in the past 48 h were tested for malaria parasites by rapid diagnostic test (CareStart malaria HRP2/pLDH [pf/pan] combo, DiaSys, Wokingham, UK). Positive tests were confirmed by microscopy. Children with a positive rapid diagnostic test or minor illness were treated by trained study nurses. A questionnaire was administered to enquire about LLIN use the night before the visit, adverse events, travel history, and visit to health facilities within the past 2 weeks. Twice a year, community health workers visited the children in their houses to verify reported LLIN use. Because of the SARS-CoV-2 pandemic, from March to September, 2020, community health workers visited cohort children at home to assess fever (axillary temperature) the day before central point study visits. Study nurses only met with children with fever or history of fever to test them for malaria. COVID-19-safe protocols were followed during these visits.

Indoor mosquito density was monitored from January, 2019, to December, 2020 (appendix p 3) using Centers for Disease Control (CDC) light traps (John W Hock, Gainesville, Florida, USA). Mosquitoes were identified morphologically, and a subsample screened for *P falciparum* circumsporozoite protein by ELISA.^15^ TaqMan PCR assays were used to differentiate *A gambiae*^16^ and *A funestus*^17^ sibling species. We assessed the intensity of resistance to α-cypermethrin, permethrin, pyriproxyfen, and chlorfenapyr using CDC bottle bioassays every year.^13^

Mosquito collections were suspended from March to May, 2020, because of the SARS-CoV-2 pandemic; to meet sample size requirements and compensate for the lost round during the high mosquito density season, the number of collection rounds was increased from four to five in 2020.

### Outcomes

The primary outcome was the prevalence of malaria infection (positive rapid diagnostic test) in children aged 6 months to 14 years at 24 months post-intervention (distribution of LLINs); secondary comparisons of this outcome were made at 12 and 18 months. The primary malaria transmission outcome was the entomological inoculation rate defined as the number of mosquito vectors tested positive for malaria over 24 months, and the primary economic outcome was cost-effectiveness based on incremental cost per disability-adjusted life-years (DALYs) averted. Secondary outcomes were the incidence of malaria cases (temperature ≥37·5°C or fever within 48 h and positive rapid diagnostic test) in children aged 6 months to 10 years, measured over 24 months of follow-up; the prevalence of moderate and severe anaemia (defined as haemoglobin concentration <8 g/dL) in children 6 months to 4 years measured at 12, 18, and 24 months; and vector density (mean number of malaria vectors collected per house per night), sporozoite rate (proportion of vectors infected with malaria parasite), and resistance to insecticides (proportion of vectors dead or sterile after exposure to insecticide). Adverse events and severe adverse events were recorded in the study cohort and in the population during cross-sectional surveys. Cause of death in the cohort was investigated by interviewing the child's guardian after receiving consent. Other secondary outcomes, including malaria prevalence, anaemia, and entomological inoculation rate at 30 and 36 months post-intervention will be published separately; data collection is ongoing.

### Statistical analysis

The required sample size was calculated using the method of Hayes and Moulton,^18^ accounting for the cluster-randomised design. For the cross-sectional surveys, we assumed a malaria prevalence of 40% in the reference group (pyrethroid-only LLINs) and a coefficient of variation of 21%.^19^ With 21 clusters per group and 50 individuals per cluster at each cross-sectional survey, the study had 80% power to detect a relative reduction in prevalence of 28% in each intervention group and the reference group at 24 months. For the cohort design, we assumed a mean of 0·85 malaria episodes per child per year in the reference group, with a between-cluster coefficient of variation of 21%. With a cohort of 35 children per cluster and 21 clusters per group, and accounting for loss to follow-up of 30% over 24 months, the study had 80% power to detect a relative reduction of 24% in the number of malaria cases per child per year in each intervention group relative to the reference group.

For the entomological survey, a mean entomological inoculation rate of 1·76 infective mosquitoes per month per household in the reference group was assumed, with a between-cluster coefficient of variation of 40%.^4^ With eight households sampled in each cluster every quarter, or 32 house-nights of collection per cluster per year, the study had 80% power to detect a relative reduction of at least 36% in monthly entomological inoculation rate between intervention and reference groups. Sample size estimates allowed for a Bonferroni-corrected significance level of 1·67% (two-sided) to account for multiple treatment group comparisons of each intervention versus control.

Statistical analyses were done with Stata (version 15). Malaria infection prevalence, malaria case incidence, anaemia, and entomological inoculation rate were compared between study group in the intention-to-treat population, defined as children allocated to LLIN type as randomised regardless of type of LLIN used. A per protocol analysis was done for malaria infection prevalence and included only children who actually used the allocated study nets. For the intention-to-treat and per protocol analyses, malaria infection prevalence at 12, 18, and 24 months was analysed by mixed-effects logistic regression. Cumulative incidence of malaria infection was analysed by mixed-effects Poisson regression with individual follow-up time specified as offset; following antimalarial treatment, 14 days follow-up time was censored to account for the prophylactic effect of the treatment. Blood slide confirmation of positive rapid diagnostic test results ensured that only those positive for parasites were counted as cases. The entomological inoculation rate was estimated as the number of sporozoite-infected mosquitoes per household per night and weighted to account for the proportion of mosquitoes processed for sporozoite detection. Differences in Anopheles density and entomological inoculation rate between study groups were estimated by mixed-effects negative binomial regression, and differences in sporozoite rate by mixed-effects logistic regression. Each model included fixed effects for time (survey timepoint or study year), study group, and time-by-study group interaction, and adjusted for the cluster-level variables used in restricted randomisation according to the prespecified analysis plan. A block-exchangeable correlation structure was used to account for the within-period and between-period intracluster correlations for all outcomes (except cumulative incidence, which was analysed using an exchangeable correlation structure).^20^ To obtain overall estimates, we re-estimated the models by omitting the time-by-study group interaction. Intervention effects were expressed as adjusted odds ratios (aORs; for malaria and anaemia prevalence), incidence rate ratios (IRRs; for malaria case incidence), and density ratios (for entomological inoculation rate), with 95% CIs obtained as least-squares mean differences from the model; a multiplicity-adjusted p value of 1·67% was used to define statistical significance. Adjusted risk ratios for malaria prevalence in the intention-to-treat population were also estimated (appendix p 14).

This trial is registered with ClinicalTrials.gov, number NCT03554616.

### Cost-effectiveness analysis

We modelled cost-effectiveness over the 2-year trial period. Malaria incidence estimates for each trial year were combined with probabilities of progression to severe disease and death on the basis of secondary sources (appendix pp 21–26). Using age-stratified malaria estimates for all countries from the Global Burden of Disease Study 2019,^21^ incidence in people older than 10 years was estimated as a function of incidence in children aged 6 months to 10 years, and deaths in people older than 10 years were estimated as a fixed ratio to modelled deaths in children aged 6 months to 10 years (appendix pp 27–28). DALYs under each strategy were estimated using standard methods, with 3% annual discounting, no age weighting, and years of life lost at death weighted to reflect the age structure of malaria deaths in Tanzania (appendix p 29).

We took a disaggregated societal perspective, meaning that we considered costs to donors, to public providers (donors and Tanzanian government combined), and to households, both separately and together. Costs of net distribution were excluded because they were identical across strategies. Costs of net procurement were obtained from The Global Fund to Fight AIDS, Tuberculosis, and Malaria. The cost per case of malaria was assumed to be the same across trial groups, with donors bearing the costs of publicly funded rapid diagnostic tests and antimalarial medicines, the Tanzanian government bearing the costs of publicly funded consultations and hospitalisations, and households bearing the costs of time spent ill and out-of-pocket payments. Details of parameter values and sources are provided in appendix (pp 23–26).

We used Monte Carlo simulation to conduct probabilistic analyses, which reflect combined uncertainty in stochastic parameters. Analyses were re-run, varying one key parameter at a time, to examine the robustness of results to plausible variations in individual parameters. A threshold analysis identified the price of each net at which cost-effectiveness conclusions would change. Analyses were done in Microsoft Excel with Visual Basic for Applications. Results are presented on the cost-effectiveness plane and compared against plausible cost-effectiveness thresholds.^22^

### Role of the funding source

The funders of the study had no role in study design, data collection, data analysis, data interpretation, or writing of the report.

## Results

Selection of villages and households was done during the census between May 11 and July 2, 2018. Enrolment of children for the cohort was done in March, 2019. The study area included 39 307 households across 72 villages. These households formed 84 clusters, which were evenly distributed between the four groups (21 [25%] clusters per group; figure 1). Between Jan 26 and 28, 2019, a total of 147 230 LLINs were distributed among households in the study area.

28 599 core-area households were eligible for cross-sectional surveys of malaria prevalence. At baseline (Aug 31 to Oct 2, 2018), characteristics of the surveyed households (n=2656) and children (n=5019) were similar between the four groups, with malaria infection detected in 1948 (44·2%) of 4403 children tested and LLIN use reported by 11 314 (60·7%) of 18 654 participants across all age groups (table 1). Across the three further cross-sectional surveys done at 12, 18, and 24 months post-intervention, a total of 11 340 households (45 per cluster per timepoint) were selected, of which 1376 (12·1%) had no children younger than 15 years, 1061 (9·4%) were absent, 199 (1·8%) refused, and 772 (6·8%) were not found or not visited (appendix p 11). From the remaining consenting households (7932 [69·9%]), 14 870 children were selected and invited for a medical appointment, of whom 14 168 attended the appointment and were tested for malaria. Of these children, five had missing malaria results and 14 163 were included in the intention-to-treat analysis (figure 1).

**Table 1:** Baseline characteristics

		**Pyrethroid-only group**	**Pyriproxyfen group**	**Chlorfenapyr group**	**Piperonyl butoxide group**
**Study clusters**					
Population					
	Overall study area	61 183	57 567	60 115	57 631
	Core cluster areas	43 877	43 266	41 748	45 020
Mean number of people per household in the study area		7·3 (3·3)	6·8 (3·0)	7·2 (3·1)	6·9 (2·8)
**Households and children (aged 6 months to 14 years) selected for baseline cross-sectional survey (September, 2018)**					
Number selected					
	Households	680	667	671	638
	Children	1295	1290	1249	1185
Median age of selected children, years		6 (3–10), N=1295	6 (3–10), N=1290	6 (3–9), N=1249	6 (3–10), N=1185
Low socioeconomic status*		210/680 (30·9%)	230/667 (34·5%)	209/671 (31·1%)	237/638 (37·1%)
Long-lasting insecticidal net use					
	In household residents of all age groups	2957/4962 (59·6%)	2813/4520 (62·2%)	2849/4803 (59·3%)	2695/4369 (61·7%)
	In selected children	831/1295 (64·2%)	839/1290 (65·0%)	781/1249 (62·5%)	751/1185 (63·4%)
Malaria infection prevalence in selected children		519/1130 (45·9%)	516/1118 (46·2%)	469/1099 (42·7%)	444/1056 (42·0%)
Anaemia† prevalence in children aged 6 months to 4 years		28/453 (6·2%)	28/500 (5·6%)	28/507 (5·5%)	20/472 (4·2%)
**Children (aged 6 months to 10 years) enrolled in cohort (March, 2019, and February, 2020)**					
Median age, years		5 (3–7), N=1523	5 (3–8), N=1523	5 (2–7), N=1495	5 (3–8), N=1527
Sex					
	Female	787/1523 (51·7%)	789/1523 (51·8%)	718/1495 (48·0%)	825/1527 (54·0%)
	Male	736/1523 (48·3%)	734/1523 (48·2%)	777/1495 (52·0%)	702/1527 (46·0%)
LLIN use at enrolment		1450/1523 (95·2%)	1473/1523 (96·7%)	1432/1495 (95·8%)	1467/1527 (96·1%)
**Entomological characteristics (August to December, 2018)**					
Mean indoor vectors per household per night		5·9 (20·9), N=335	4·2 (18·0), N=334	2·8 (17·5), N=334	1·9 (7·0), N=337
Sporozoite rate		30/680 (4·4%)	19/570 (3·3%)	7/318 (2·2%)	11/367 (3·0%)
Mean entomological inoculation rate per household per night		0·35 (0·72), N=331	0·11 (0·34), N=328	0·04 (0·20), N=332	0·07 (0·26), N=326
*Anopheles funestus* proportion		1869/1982 (94·3%)	1331/1401 (95·0%)	904/948 (95·4%)	596/642 (92·8%)

Data are n, mean (SD), n/N (%), or median (IQR). LLIN=long-lasting insecticidal net.

* Proportion of households in the poorest tercile based on the wealth index of the entire study area.

† Defined as haemoglobin concentration <8 g/dL.

For the cohort, 2982 children aged 6 months to 10 years were recruited in March, 2019, and 3371 in February, 2020, giving a total follow-up time of 5565·5 child-years. Baseline characteristics are reported in table 1. Loss to follow-up was similar between groups (figure 1).

Between Aug 20 and Dec 13, 2018, baseline mean malaria vector density per household per night ranged from 1·9 (SD 7·0) to 5·9 (20·9) across study groups (n=1340 households), and 4700 (94·5%) of 4973 Anopheles collected were identified as *A funestus*. Across nine further collection rounds, entomological data were collected in 6043 households (figure 1; appendix p 18).

3 months after LLIN distribution, 3155 (72·1%) of 4378 of study participants surveyed reported using the assigned study LLINs the previous night; this proportion was similar between study groups (appendix p 4). Most of the other nets owned were standard LLINs distributed during antenatal visits and through schools before our trial. Use of study LLINs decreased to 8694 (40·9%) of 21 246 over 2 years, at different rates between intervention groups; at 24 months, use of the assigned study LLIN was reported in 2488 (49·5%) of 5029 participants in the pyrethroid-only group, 2087 (38·3%) of 5455 in the pyriproxyfen group, 2585 (46·4%) of 5576 in the chlorfenapyr group, and 1534 (29·6%) of 5186 in the piperonyl butoxide group. Use of any LLINs (including non-study LLINs) ranged from 76·5% to 82·6% at 24 months (appendix pp 4–7).

The proportion of study LLINs that were torn (defined as hole area ≥790 cm^2^)^23^ was 86 (28%) of 303 in the pyrethroid-only group, 109 (39%) of 282 in the pyriproxyfen group, 96 (34%) of 284 in the chlorfenapyr group, and 81 (43%) of 188 in the piperonyl butoxide group (appendix p 8). Chemical analysis on 30 nets per group showed that the active ingredient concentration in each type of LLIN met the specification criteria when new. At 24 months, partner active ingredient retention was 28% for pyriproxyfen, 18% for chlorfenapyr, and 30% for piperonyl butoxide (appendix pp 9–10).

In the intention-to-treat analysis at 24 months (table 2), no statistically significant reduction in the prevalence of malaria infection was observed in the pyriproxyfen group (472 [37·5%] of 1258 children; aOR 0·79 [95% CI 0·54–1·17], p=0·2354) or the piperonyl butoxide group (512 [40·7%] of 1259; 0·99 [0·67–1·45], p=0·9607) relative to the pyrethroid-only reference group (549 [45·8%] of 1199); however, prevalence was significantly lower in the chlorfenapyr group (326 [25·6%] of 1272; 0·45 [0·30–0·67], p=0·0001) than in the pyrethroid-only group. Similarly, at 12 months, a statistically significant reduction was observed only in the chlorfenapyr group and not in the pyriproxyfen or piperonyl butoxide groups, whereas no group showed a significant reduction relative to the pyrethroid-only group at 18 months (table 2). Per protocol analysis results were similar (table 2). Risk ratios for the intention-to-treat analysis are reported in the appendix (p 14). A sensitivity analysis showed that the results were unchanged if adjustment for differences in entomological outcomes at baseline were made. The within-period intracluster correlation coefficient (ICC) was 0·059, with a between-period ICC of 0·022 and cluster autocorrelation coefficient (CAC) of 0·375.

**Table 2:** Malaria infection prevalence in children (aged 6 months to 14 years) at 12, 18, and 24 months after intervention

	**Intention-to-treat analysis**			**Per protocol analysis**		
	Malaria prevalence	aOR (95% CI)*	p value†	Malaria prevalence	aOR (95% CI)*	p value†
**Pyrethroid-only group**						
12 months	350/1123 (31·2%)	1 (ref)	..	203/676 (30·0%)	1 (ref)	..
18 months	642/1227 (52·3%)	1 (ref)	..	330/651 (50·7%)	1 (ref)	..
24 months	549/1199 (45·8%)	1 (ref)	..	250/569 (43·9%)	1 (ref)	..
**Pyriproxyfen group**						
12 months	232/1069 (21·7%)	0·69 (0·48–1·04)	0·0754	120/621 (19·3%)	0·59 (0·37–0·95)	0·0286
18 months	583/1153 (50·6%)	0·98 (0·67–1·44)	0·9184	245/541 (45·3%)	0·81 (0·52–1·27)	0·3628
24 months	472/1258 (37·5%)	0·79 (0·54–1·17)	0·2354	151/441 (34·2%)	0·71 (0·45–1·13)	0·1510
**Chlorfenapyr group**						
12 months	176/1126 (15·6%)	0·47 (0·31–0·71)	0·0003	108/716 (15·1%)	0·45 (0·28–0·72)	0·0009
18 months	509/1246 (40·9%)	0·66 (0·45–0·97)	0·0365	254/656 (38·7%)	0·63 (0·40–0·97)	0·0382
24 months	326/1272 (25·6%)	0·45 (0·30–0·67)	0·0001	131/598 (21·9%)	0·39 (0·25–0·63)	0·0001
**Piperonyl butoxide group**						
12 months	206/1071 (19·2%)	0·65 (0·44–0·99)	0·0421	128/627 (20·4%)	0·77 (0·49–1·22)	0·2697
18 months	502/1160 (43·3%)	0·76 (0·52–1·12)	0·1699	192/469 (40·9%)	0·71 (0·45–1·11)	0·1334
24 months	512/1259 (40·7%)	0·99 (0·67–1·45)	0·9607	139/356 (39·0%)	0·97 (0·60–1·55)	0·8891

Prevalence data are n/N (%). For time-by-study group interaction, p_interaction_=0·0738 in intention-to-treat analysis and p_interaction_=0·2267 in per protocol analysis. Each intervention group is compared against the pyrethroid-only group for the same timepoint. aOR=adjusted odds ratio.

* Adjusted for baseline cluster-level variables used in restricted randomisation.

† p<0·017 was considered statistically significant after Bonferroni correction.

Consistent with malaria cross-sectional prevalence results, in the cohort of children aged 6 months to 10 years, malaria clinical case incidence over 24 months of follow-up (1380·7 to 1398·5 child-years per group) was 0·46 per child-year in the pyrethroid-only reference group, 0·42 per child-year in the pyriproxyfen group (IRR 0·99 [95% CI 0·66–1·50], p=0·9801), 0·23 per child-year in the chlorfenapyr group (0·56 [0·37–0·86], p=0·0072), and 0·33 per child-year in the piperonyl butoxide group (0·92 [0·61–1·38], p=0·6809; table 3). The intervention effect on malaria incidence varied significantly by time of follow-up (p=0·0001 for time-by-study group interaction), with the strongest difference observed in year 1 in the chlorfenapyr group (IRR 0·46 [95% CI 0·28–0·74], p=0·0016) and the piperonyl butoxide group (0·53 [0·33–0·85], p=0·0090). No significant difference in moderate and severe anaemia was observed in any of the intervention groups at any time point (appendix pp 17).

**Table 3:** Malaria case incidence in children (aged 6 months to 10 years)

	**Number of clinical episodes**	**Follow-up time, child-years**	**Incidence per child per year**	Adjusted incidence rate ratio (95% CI)*	**p value**†
**Pyrethroid-only group**					
Year 1	194	605·1	0·32	1 (ref)	..
Year 2	449	793·4	0·57	1 (ref)	..
Overall	643	1398·5	0·46	1 (ref)	..
**Pyriproxyfen group**					
Year 1	161	604·5	0·27	0·94 (0·60–1·48)	0·8029
Year 2	418	787·4	0·53	1·02 (0·67–1·55)	0·9235
Overall	579	1391·9	0·42	0·99 (0·66–1·50)	0·9801
**Chlorfenapyr group**					
Year 1	79	603·8	0·13	0·46 (0·28–0·74)	0·0016
Year 2	248	790·6	0·31	0·61 (0·40–0·94)	0·0250
Overall	327	1394·4	0·23	0·56 (0·37–0·86)	0·0072
**Piperonyl butoxide group**					
Year 1	79	591·8	0·13	0·53 (0·33–0·85)	0·0090
Year 2	381	788·9	0·48	1·11 (0·73–1·67)	0·6306
Overall	460	1380·7	0·33	0·92 (0·61–1·38)	0·6809

For time-by-study group interaction, p_interaction_=0·0001. Each intervention group is compared to the pyrethroid group for the same timepoint.

* Adjusted for baseline cluster-level variables used in restricted randomisation.

† p<0·017 was considered statistically significant after Bonferroni correction.

Five deaths among cohort children were reported to the data safety monitoring board (figure 1). Three deaths were from drowning, one was due to severe malaria, and one due to pneumonia, all of which were judged to be unrelated to the study interventions. Side-effects related to use of standard pyrethroid-only LLINs were reported in 90 (44·1%) of 204 participants at 3 months post-distribution, 167 (10·1%) of 1647 at 12 months, two (0·1%) of 1579 at 18 months, and 11 (0·7%) of 1683 at 24 months. Similarly, for those using pyriproxyfen LLINs, side-effects were reported in 80 (38·8%) of 206 participants at 3 months, 143 (9·3%) of 1543 at 12 months, none of 1425 at 18 months, and seven (0·4%) of 1692 at 24 months. Lower frequencies of side-effects were reported in the piperonyl butoxide group (17 [8·5%] of 199) and the chlorfenapyr group (17 [8·5%] of 199) at 3 months (appendix p 12). Skin irritation or paraesthesia was the most commonly reported side-effect in all groups, reported in 497 (83·1%) of 598 participants who reported side-effects (appendix p 13). No serious or severe side-effects were reported.

Entomological inoculation rate was significantly reduced in the chlorfenapyr group in both years (adjusted density ratio 0·15 [95% CI 0·07–0·31], p<0·0001 [for years 1 and 2 combined]) and in the piperonyl butoxide group in year 1 only (0·27 [0·09–0·79], p=0·0167) compared with that in the pyrethroid-only group. No significant difference was observed in the pyriproxyfen group compared with the pyrethroid-only group at any timepoint (table 4).

**Table 4:** Entomological outcomes

	**Vector density**					**Sporozoite rate**					**Entomological inoculation rate**		
	Number of households analysed	Number of female *Anopheles*	Density per household per night	Adjusted density ratio (95% CI)*	p value†	Number of sporozoite-positive female *Anopheles*	Number of female *Anopheles* tested	Sporozoite rate	Adjusted odds ratio (95% CI)*	p value†	Mean per household per night	Adjusted density ratio (95% CI)*‡	p value†
**Pyrethroid-only group**													
Year 1	670	1658	2·47	1 (ref)	..	14	1009	1·4%	1 (ref)	..	0·038	1 (ref)	..
Year 2	838	6352	7·58	1 (ref)	..	47	2422	1·9%	1 (ref)	..	0·093	1 (ref)	..
Overall	1508	8010	5·31	1 (ref)	..	61	3431	1·8%	1 (ref)	..	0·069	1 (ref)	..
**Pyriproxyfen group**													
Year 1	672	795	1·18	0·62 (0·36–1·08)	0·0899	12	633	1·9%	1·93 (0·74–5·01)	0·1778	0·020	0·48 (0·19–1·20)	0·1182
Year 2	840	7633	9·09	0·90 (0·56–1·44)	0·6560	34	2144	1·6%	0·95 (0·52–1·73)	0·8683	0·097	0·82 (0·40–1·67)	0·5800
Overall	1512	8428	5·57	0·77 (0·52–1·14)	0·1947	46	2777	1·7%	1·15 (0·68–1·93)	0·6098	0·064	0·73 (0·40–1·34)	0·3108
**Chlorfenapyr group**													
Year 1	671	435	0·65	0·33 (0·19–0·58)	0·0001	2	340	0·6%	0·63 (0·13–3·07)	0·5625	0·003	0·08 (0·02–0·40)	0·0020
Year 2	840	5058	6·02	0·51 (0·32–0·83)	0·0064	12	1526	0·8%	0·43 (0·20–0·93)	0·0316	0·015	0·18 (0·08–0·40)	<0·0001
Overall	1511	5493	3·64	0·43 (0·29–0·64)	<0·0001	14	1866	0·8%	0·48 (0·24–0·95)	0·0352	0·010	0·15 (0·07–0·31)	<0·0001
**Piperonyl butoxide group**													
Year 1	672	500	0·74	0·42 (0·24–0·73)	0·0023	6	447	1·3%	1·04 (0·34–3·24)	0·9418	0·010	0·27 (0·09–0·79)	0·0167
Year 2	840	3914	4·66	0·64 (0·40–1·03)	0·0678	33	1702	1·9%	0·84 (0·46–1·53)	0·5618	0·059	0·67 (0·34–1·31)	0·2427
Overall	1512	4414	2·92	0·54 (0·37–0·80)	0·0020	39	2149	1·8%	0·89 (0·52–1·53)	0·6747	0·038	0·56 (0·32–0·98)	0·0429

For time-by-study group interaction, p_interaction_=0·5173 for vector density, p_interaction_=0·6454 for sporozoite rate, and p_interaction_=0·5900 for entomological inoculation rate.

* Adjusted for baseline cluster-level variables used in restricted randomisation.

† p<0·017 was considered statistically significant after Bonferroni correction.

‡ Weighted to account for the proportion of mosquitoes sampled to be tested for sporozoites.

Pyrethroid resistance was high in *A funestus*, with mortality below 85% after exposure to 10 times diagnostic concentrations of α-cypermethrin or permethrin. Exposure to pyriproxyfen sterilised only 127 (24%) of 536 of *A funestus* and 23 (21%) of 112 *A gambiae* sensu lato. No resistance was observed against chlorfenapyr insecticide (appendix pp 19–20).

**Figure 2:**
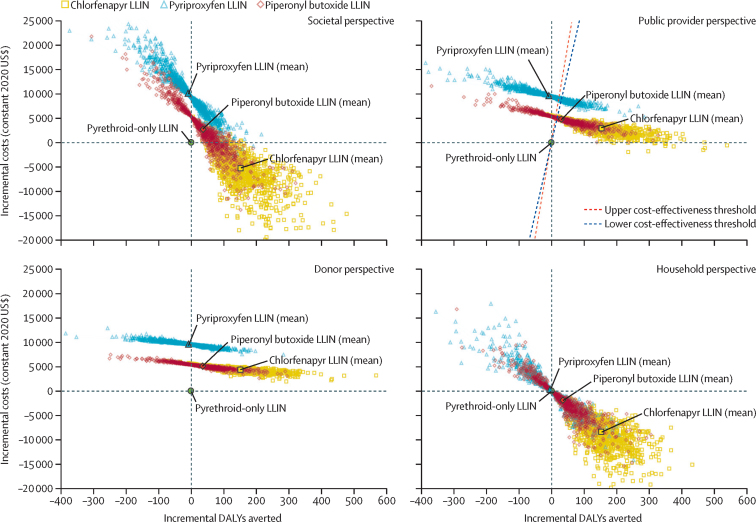
Cost-effectiveness of dual-active-ingredient LLINs relative to pyrethroid-only LLINs over a 2-year period Cost-effectiveness planes are shown separately for societal, donor, public provider, and household perspectives. The public provider perspective combines costs borne by donors with costs borne by the public health service in providing LLINs and malaria diagnoses and treatments. Each data point reflects a single iteration in the Monte Carlo simulation; 1000 iterations were conducted. LLIN=long-lasting insecticidal net. DALYs=disability-adjusted life-years.

Consistent with malaria case incidence estimates, compared with pyrethroid-only LLINs, chlorfenapyr LLINs were estimated to avert the most DALYs (mean 152 DALYs averted [SD 72] per 10 000 total population), followed by piperonyl butoxide LLINs (37 DALYs averted [72] per 10 000 population), while pyriproxyfen LLINs incurred 9 more DALYs [71] per 10 000 population than did pyrethroid-only LLINs (figure 2; appendix p 31). Pyrethroid-only LLINs were the least costly to procure (US$2·07 per net), followed by piperonyl butoxide LLINs ($2·98), chlorfenapyr LLINs ($3·02), and pyriproxyfen LLINs ($3·68). However, when the costs of malaria diagnosis and treatment were included, chlorfenapyr LLINs were the least costly of the dual-active-ingredient LLINs from all perspectives (incremental cost $2894 [SD 1129] per 10 000 population compared with pyrethroid-only LLINs over 2 years, from the provider perspective) followed by piperonyl butoxide LLINs ($4816 [1360]), with pyriproxyfen LLINs ($9621 [1327]) consistently being the most expensive strategy (figure 2; appendix p 31). From societal and household perspectives, chlorfenapyr LLINs would be more effective and less costly than all other LLINs (including standard pyrethroid-only LLINs) over a 2-year period, making them the dominant choice. From public provider and donor perspectives, chlorfenapyr LLINs would be the most cost-effective of the three dual-active-ingredient LLINs, costing an additional $19 (95% uncertainty interval 1–105) to public providers or $28 (11–120) to donors per DALY averted relative to pyrethroid-only LLINs—well below plausible cost-effectiveness thresholds ($292–393 per DALY averted; appendix p 23). Although piperonyl butoxide LLINs were less effective and more costly than chlorfenapyr LLINs from all perspectives, they would be cost-effective relative to pyrethroid-only LLINs; they were estimated to cost an additional $130 to public providers ($136 to donors) per DALY averted, although the uncertainty interval varied from a low of $12 ($22 to donors) per DALY averted up to –$59 (–$58 to donors), indicating that they could incur additional costs and also worsen health outcomes (appendix p 31). Findings were robust to plausible variations in key parameters (appendix pp 32–34) for this setting.

## Discussion

In this trial assessing three types of dual-active-ingredient LLINs in an area of Tanzania with high pyrethroid resistance among *Anopheles* mosquitoes, we found that only the chlorfenapyr LLINs provided significantly better protection over 2 years than did pyrethroid LLINs; children aged 6 months to 14 years had 55% lower odds of having malaria 2 years after LLIN distribution, and children aged 6 months to 10 years had 44% lower malaria incidence over the 2 years. The entomological inoculation rate, an indicator of malaria transmission, was also 85% lower in the chlorfenapyr group than in the pyrethroid-only group, arising from reduced vector population density and longevity. Added protection provided by piperonyl butoxide LLINs lasted for only 1 year, with clinical malaria incidence in children nearly halved and a 73% reduction in entomological inoculation rate, but with no evident effect on malaria prevalence. Although the point estimates for malaria prevalence and entomological inoculation rate were lower in the pyriproxyfen LLIN group compared with the pyrethroid-only reference group during the first year, these differences were not statistically significant. Chlorfenapyr LLINs were the most effective and cost-effective of the dual-active-ingredient LLINs; in this context, they would be less costly than even standard pyrethroid LLINs from societal and household perspectives, and well within plausible cost-effectiveness thresholds for public providers. Piperonyl butoxide LLINs were more cost-effective than pyrethroid-only nets, but unlikely to be cost-saving from a societal perspective.

This trial provides the first evidence of the additional effectiveness and cost-effectiveness of chlorfenapyr LLINs relative to pyrethroid LLINs for malaria control. Several small-scale studies had already shown the efficacy of these nets against entomological outcomes, reporting higher mortality in resistant *A gambiae*^12^ and *A funestus*^24^ compared with pyrethroid LLINs. In these experimental hut studies, intensive net washing did not significantly reduce efficacy. In our trial, the substantial reduction in vector density and sustained effect on malaria outcomes over 2 years confirm the long-lasting effectiveness of chlorfenapyr LLINs. This effectiveness was achieved despite decreases in LLIN use and chlorfenapyr concentration after 24 months, which could explain the smaller reductions in malaria incidence and entomological inoculation rate in the second year. While our 2-year follow-up meets WHO requirements for recommendation, ongoing monitoring is needed to understand whether chlorfenapyr LLINs maintain their effectiveness for 3 years—the usual interval between LLIN distribution campaigns—and to inform the replacement frequency of these nets.

Kawada and colleagues^25^ hypothesised that the addition of pyriproxyfen to an LLIN would sterilise vectors that survived exposure to the pyrethroid, reducing progeny, overall vector density, and ultimately malaria transmission. A single-year stepped-wedge RCT in Burkina Faso with a different brand of pyriproxyfen LLIN found a 12% reduction in clinical malaria incidence and a 49% reduction in entomological inoculation rate compared with standard LLINs.^10^ By contrast, the reductions observed in our trial for the pyriproxyfen LLIN were not statistically significant compared with pyrethroid LLINs, nor sustained over time. Rapid reduction in use of pyriproxyfen LLINs could partly explain these results. Low bioavailability due to slow diffusion of active ingredient from the inner core of the net fibres might also limit sustained effects on mosquito fertility rates, despite the relatively high pyriproxyfen reservoir retained in the net fibre, as was observed in other studies.^8^, ^9^, ^26^ Additionally, in our trial, pyriproxyfen resistance might have compromised the net's effectiveness, as indicated by only a quarter of vectors exposed to pyriproxyfen showing signs of ovarian development disruptions. Similar mechanisms, involving elevated concentrations of cytochrome P450s, drive resistance to pyrethroid and pyriproxyfen in Anopheles populations,^27^ raising concerns about using pyriproxyfen LLIN as a resistance-management tool.

The piperonyl butoxide LLIN (Olyset Plus) showed additional effectiveness compared with the pyrethroid-only LLIN over a shorter period (12 months) than in previous RCTs (21–25 months).^28^ In an earlier RCT in Tanzania,^4^ use of the same brand of piperonyl butoxide LLIN was 50% in the second year, which could explain why efficacy was sustained for longer in that study than in the current study, in which use of piperonyl butoxide LLINs declined more quickly and was only around 30% after 2 years. Use of piperonyl butoxide LLINs was also lower than that of study LLINs in the other groups in this study (40–50%). Of the piperonyl butoxide LLINs still in use after 1 and 2 years, the proportion of nets that were torn was greater than that of the other LLINs. Piperonyl butoxide LLIN use declined in line with the textile condition of the nets; users might have perceived them as too damaged to protect against mosquitoes due to the rapid accumulation of holes. As torn piperonyl butoxide LLINs can still provide better personal protection than pyrethroid LLINs,^29^ encouraging the community to repair and continue using these nets could partly reduce their loss. However, given the poor textile durability of all three dual-active-ingredient LLINs evaluated, improvement in their physical integrity should be a priority. Although dual-active-ingredient LLINs are more expensive than pyrethroid-only LLINs, we have shown that they can be highly cost-effective and even cost-saving compared with pyrethroid-only LLINs if they provide sufficient additional protection over a sustained period.

Our trial's combined assessment of epidemiological, entomological, economic, and LLIN durability outcomes significantly advances the evidence base on all three classes of dual-active-ingredient LLIN currently available for malaria control. Nonetheless, limitations remain. First, the rapid decrease in use of study LLINs, within a context of high overall net use, might partly explain the relative lack of effectiveness of piperonyl butoxide LLINs and pyriproxyfen LLINs over the 2 years compared with pyrethroid-only LLINs. Second, the size of the cluster buffer areas, despite being maximised given the study area, might not have totally prevented contamination of effects between intervention groups. Any residual overspill would have biased results towards the null. Third, if chlorfenapyr LLINs maintain their relative effectiveness in a third year of use, our findings will have underestimated the cases averted and associated reductions in malaria treatment costs.

This study represents the first time, to our knowledge, since the initial deployment of pyrethroid-treated nets 40 years ago that a new class of insecticide (the pyrrole chlorfenapyr) has proven safe and suitable for use on LLINs, with demonstrable effectiveness in controlling malaria. Unlike all other insecticides used in malaria control, chlorfenapyr has a non-neural mode of action and no known resistance in mosquitoes. However, caution is needed; massive scale-up of standard pyrethroid-only LLINs 10–20 years ago led to the spread of pyrethroid resistance.^1^, ^30^ Whereas the WHO global plan for insecticide resistance management in malaria vectors was developed after pyrethroid resistance was already widespread, the challenge now is to preserve chlorfenapyr's effectiveness by developing rational resistance management strategies, including co-deployment of chlorfenapyr LLINs with other new active ingredients entering the market for indoor residual spraying and LLINs. With barely 20% coverage of piperonyl butoxide LLINs in sub-Saharan Africa, substantial gains could be made with wider deployment of these nets; however, manufacturers need to improve LLIN textile durability, incentivised by global procurement approaches that focus not only on LLIN price, but also on performance and durability. Further development work is needed to optimise the effectiveness of dual-active-ingredient LLINs and to understand their strategic role in malaria control.

### Data sharing

Deidentified data and data dictionary will be made available at the end of the third year of trial follow-up upon reasonable request to the corresponding author.

## Declaration of interests

We declare no competing interests.
