# Supplementary material for: Effectiveness and cost-effectiveness against malaria of three types of dual-active-ingredient long-lasting insecticidal nets (LLINs) compared with pyrethroid-only LLINs in Tanzania: a four-arm, cluster-randomised trial
Source: Lancet. 2022 Mar 26;399(10331):1227–41. doi: 10.1016/S0140-6736(21)02499-5 (PMC8971961; doi:10.1016/S0140-6736(21)02499-5)
Supplement: Supplementary appendix [file mmc1.pdf]

THE LANCET

Supplementary appendix

This appendix formed part of the original submission and has been peer reviewed. We post it as supplied by the authors.

Supplement to: Mosha JF, Kulkarni MA, Lukole E,et al. Effectiveness and cost-effectiveness against malaria of three types of dual-active-ingredient long-lasting insecticidal nets (LLINs) compared with pyrethroid-only LLINs in Tanzania: a four-arm, cluster-randomised trial. *Lancet* 2022; **399:** 1227–41.

Effectiveness and cost-effectiveness against malaria of three types of dual-active-ingredient long-lasting insecticidal nets (LLINs) compared with pyrethroid-only LLINs in Tanzania: a four-arm, cluster-randomised trial

SUPPLEMENTARY APPENDIX

Contents

I. STUDY TIMETABLE 3

II. USE OF NETS, PHYSICAL INTEGRITY, AND CHEMICAL CONTENTS 4

S1: LLIN coverage 4
S2: Use of LLINs 6
S3: Type of nets owned 7
S4: Physical condition of netting material in the study LLINs 8
S5: Concentration of active ingredient (insecticide and PBO synergist) 9
S6: Reduction in pyrethroid and second AI content over time 10

III. EPIDEMIOLOGY. 11

S7: Consent during malaria prevalence cross-sectional surveys 11
S8: Side effects from using the study nets 12
S9: Type of side effects 13
S10: Secondary analysis of malaria prevalence: Odds ratio and relative risk 14
S11: Incidence sensitivity analysis 15
S12: Monthly malaria cases 16
S13: Anaemia prevalence in children from 6 months to 5 years old 17

IV. ENTOMOLOGY 18

S14: Consent during entomological data collection 18
S15. Insecticide resistance results 19
S16: *Anopheles* mortality 30 minutes after exposure to different doses of insecticides 20

V. ECONOMIC EVALUATION 21

S17. Decision tree 21
S18. Model structure 22
S19. Input parameters for the cost-effectiveness analysis 23
S20. Modelling of cases and deaths in persons > 10 years 27
S21. Relationship between age and malaria incidence and death rates 28
S22. Calculation of disability-adjusted life-years (DALYs) 29
S23. Cases, deaths, DALYs, and costs by net type, year, and age group 30
S24. Mean costs, effects, and incremental cost-effectiveness ratios 31
S25. Economic evaluation sensitivity analysis results 32

VI. REFERENCES 35

I. Study timetable

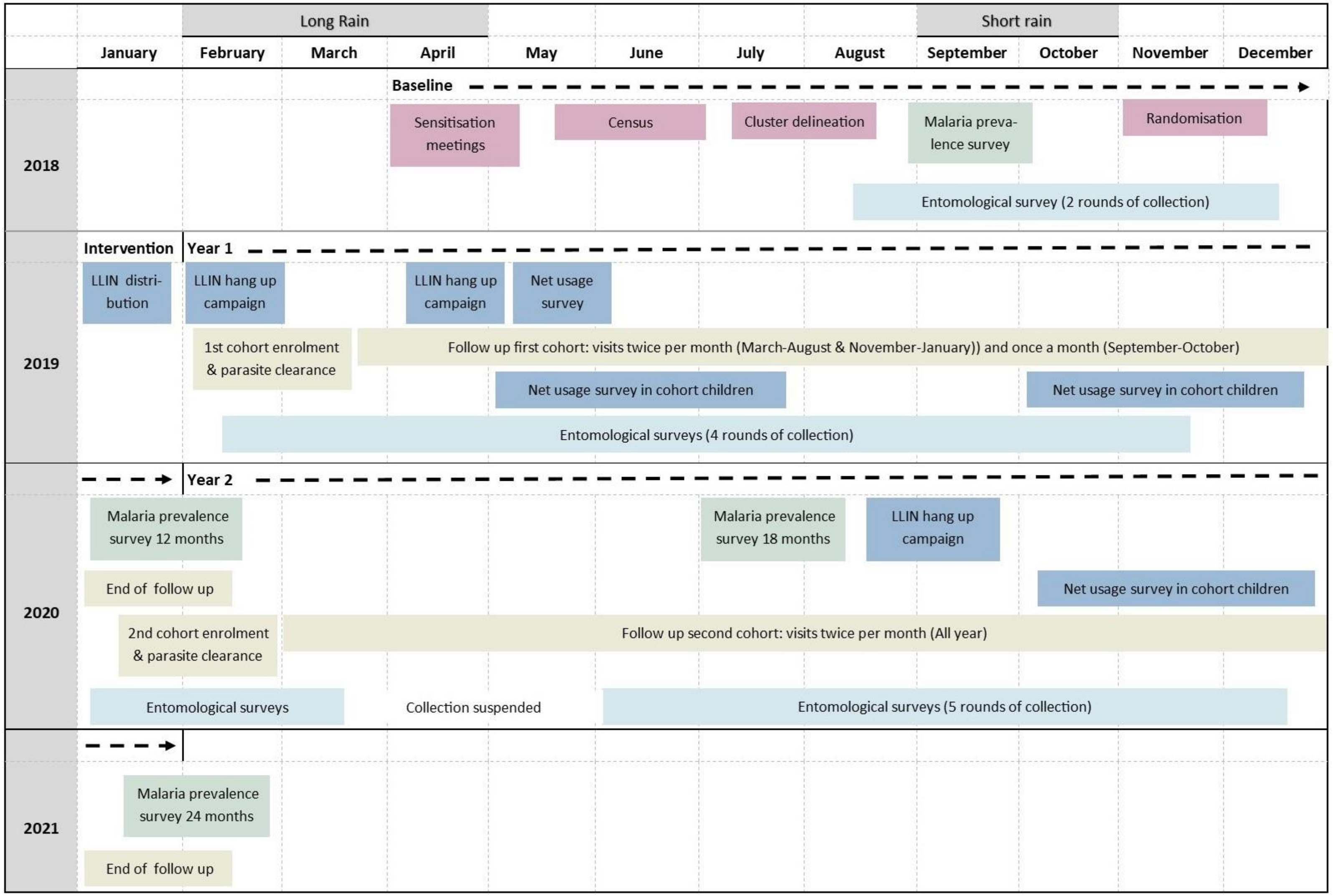

II. Use of nets, physical integrity, and chemical contents

S1: LLIN coverage

	Standard pyrethroid LLIN arm		Pyriproxyfen LLIN arm		Chlorfenapyr LLIN arm		PBO LLIN arm	
	Any LLIN	Study LLIN	Any LLIN	Study LLIN	Any LLIN	Study LLIN	Any LLIN	Study LLIN
**Proportion of households with at least one net (Ownership) % (n/N)**								
3 months	99.4 (167/168)	98.2 (165/168)	100 (169/169)	98.8 (167/169)	100 (166/166)	97.0 (161/166)	99.4 (164/165)	97 (160/165)
12 months	99.7 (641/643)	93.5 (601/643)	99.2 (614/619)	92.2 (571/619)	99.4 (681/685)	92.6 (634/685)	99.8 (615/616)	90.9 (560/616)
18 months	99.3 (670/675)	89.5 (604/675)	98.6 (648/657)	85.8 (564/657)	98.7 (680/689)	88.7 (611/689)	98.7 (680/689)	82.7 (525/635)
24 months	99.1 (656/662)	83.8 (555/662)	98.5 (679/689)	77.6 (535/689)	98.4 (682/693)	84.4 (585/693)	98.0 (678/692)	65.8 (455/692)
**Proportion of households with at least one net for every two people (HH Access) % (n/N)**								
3 months	86.3 (145/168)	60.7 (102/168)	85.2 (144/169)	62.1 (105/169)	81.9 (136/166)	55.4 (92/166)	84.8 (140/165)	64.8 (107/165)
12 months	88.6 (570/643)	61.7 (397/643)	88.0 (545/619)	58.8 (364/619)	83.4(552/662)	59.4 (393/662)	83.9 (517/616)	55.4 (341/616)
18 months	80.4 (543/675)	48.1 (325/675)	76.9 (505/657)	42.5 (279/657)	80.3 (553/689)	48.5 (334/689)	70.7 (449/635)	36.5 (232/635)
24 months	73.3 (485/662)	44.7 (296/662)	65.5 (451/689)	31.8 (219/689)	68.8 (477/693)	41.4 (287/693)	65.3 (452/692)	28.3 (196/692)
**Average number of nets per Household mean (sd)**								
3 months	4.7 (2.2)	3.4 (1.7)	4.6 (3.1)	3.2 (2.0)	4.8 (2.2)	3.3 (1.8)	4.4 (2.3)	3.2 (1.8)
12 months	3.9 (1.9)	2.7 (1.5)	3.8 (1.7)	2.6 (1.5)	3.9 (1.8)	2.7 (1.6)	3.6 (1.7)	2.3 (1.5)
18 months	3.6 (1.9)	2.3 (1.5)	3.3 (1.7)	2.0 (1.4)	3.6 (1.9)	2.3 (1.5)	3.2 (1.7)	1.7 (1.3)
24 months	3.3 (1.8)	1.9 (1.4)	3.1 (1.6)	1.6 (1.33)	3.3 (1.7)	1.9 (1.4)	2.9 (1.5)	1.2 (1.2)
**Proportion of participants reporting using a net the night before % (n/N)**								
3 months	88.0 (994/1130)	76.8 (868/1130)	85.1 (939/1103)	69.3 (764/1103)	84.4 (928/1099)	68.4 (752/1099)	85.8 (897/1046)	73.7 (771/1046)
12 months	85.4 (3953/4627)	61.6 (2848/4627)	83.1 (3621/4358)	60.7 (2646/4358)	84.9 (4102/4833)	65.3 (3157/4833)	86.8 (3688/4247)	59.0 (2506/4247)
18 months	78.6 (3925/4991)	52.2 (2606/4991)	75.1 (3487/4645)	45.7 (2124/4645)	74.8 (3885/5194)	51.5 (2676/5194)	74.4 (3445/4633)	40.7 (1885/4633)
24 months	82.6 (4155/5029)	49.5 (2488/5029)	77.6 (4231/5455)	38.3 (2087/5455)	77.0 (4291/5576)	46.4 (2585/5576)	76.5 (3966/5186)	29.6 (1534/5186)
**Proportion of selected children reporting using a net the night before % (n/N)**								
12 months	86.2 (1034/1200)	59.5 (714/1200)	83.3 (957/1149)	57.4 (659/1149)	85.9 (1057/1230)	64.0 (787/1230)	87.9 (1000/1138)	57.9 (659/1138)
18 months	80.2 (1024/1277)	52.6 (671/1277)	78.2 (948/1213)	46.7 (566/1213)	76.3 (978/1281)	52.4 (671/1281)	76.7 (924/1205)	40.1 (483/1205)
24 months	82.7 (1030/1246)	47.1 (587/1246)	79.2 (1035/1306)	35.3 (461/1306)	78.3 (1030/1316)	46.7 (615/1316)	79.4 (1039/1309)	28.3 (370/1309)

Proportion of selected cohort children reporting using a net the night before % (n/visits)

Year 1 cohort	88.9 (11095/12474)	88.4 (11032/12474)	88.3 (11009/12474)	87.8 (10951/12472)	87.0 (10836/12458)	86.6 (10,794/12458)	86.5 (10755/12428)	86.3 (10720/12428)
Year 2 cohort	91.8 (9433/10276)	90.9 (9340/10276)	91.9 (9443/10271)	90.7 (9317/10271)	92.0 (9355/10166)	90.8 (9227/10166)	90.3 (9236/10224)	87.5 (8942/10224)

S2: Use of LLINs

The figures show the proportion of household participants (all age groups) reporting that the night before the visit, they used either: (a) the correct study LLIN, or (b) any LLIN (includes study LLIN and other LLIN).

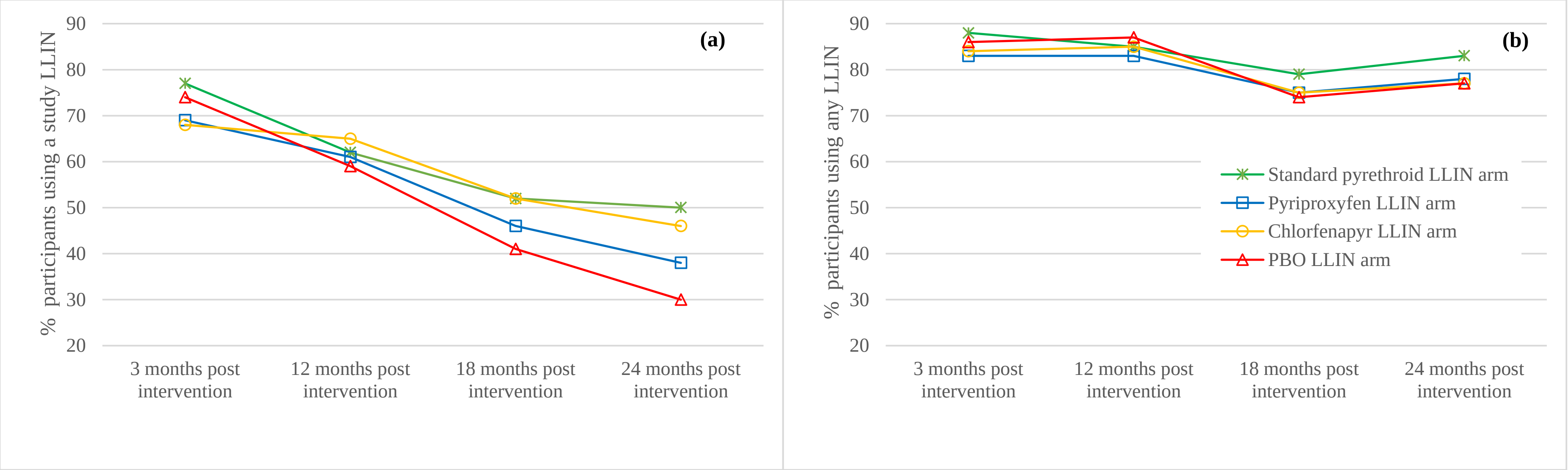

S3: Type of nets owned

The figure shows the proportion of different types of nets found in households. “Study net” includes all the nets distributed at the start of the trial by the project. At baseline, 59% of all nets found in households were Olyset™ Net (standard pyrethroid-only LLIN), which had been distributed during the previous universal coverage campaign in 2015 and through antenatal care. PermaNet® 2.0 (standard pyrethroid LLIN) accounted for 33% of the nets; this brand of nets had been distributed during annual school net distribution. During the trial, school net distribution was suspended and new LLINs (Olyset Net and Permanet 2.0 in 2019 and Olyset Plus (PBO LLIN) in 2020) were only distributed at health facilities to pregnant women during antenatal visits. At 3 months’ time point, the mean number of LLIN owned per households was 4.6 (3.4 study LLIN and 1.2 other net) for an average household size of 7 people. At this time point, most of the other nets (none study LLINs) owned were PermaNet 2 and Olyset Net and were obtained before the study net trial distribution. Mean number of nets at 24 months was 3.2 with an average of 1.7 study LLINs and 1.5 other nets (see S1).

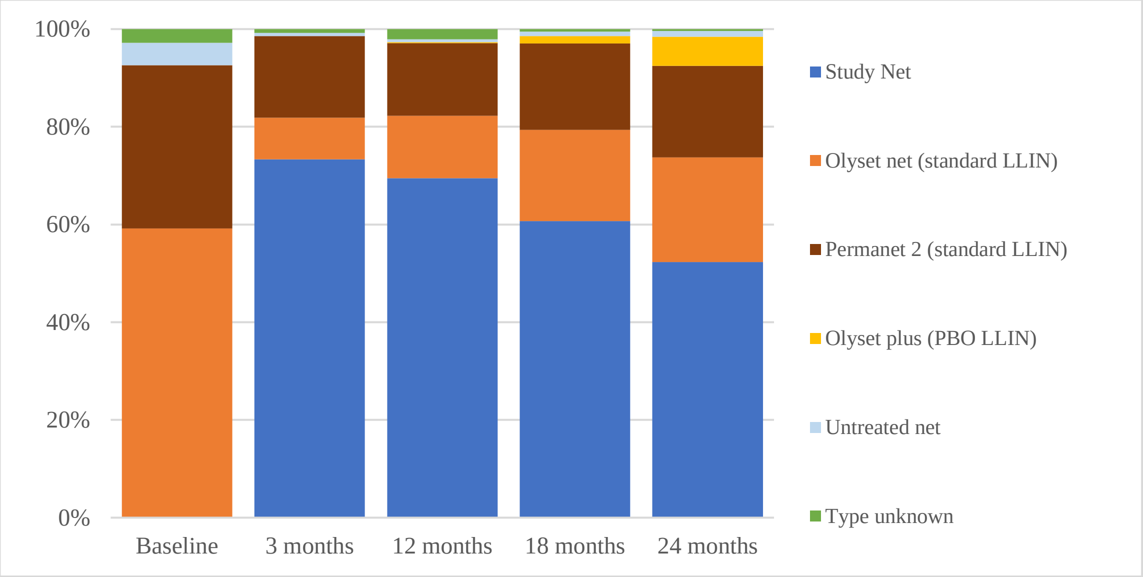

S4: Physical condition of netting material in the study LLINs

The table shows the physical condition of netting material in the study LLINs inspected after 12 and 24 months of use during household cross-sectional surveys.

	Standard pyrethroid LLIN	Pyriproxyfen LLIN	Chlorfenapyr LLIN	PBO LLIN
**12 months post intervention**				
Net inspected, N	286	243	342	245
% good condition* (n), 95%CI	68.2 (195), 62.5-73.3	69.1 (168), 63.0-74.6	70.2 (240), 65.1-74.8	49.8 (122), 43.5-56.1
% acceptable condition* (n), 95%CI	18.5 (53), 14.4-23.5	14.0 (34), 10.2-19.0	18.1 (62), 14.4-22.6	21.6 (53), 16.9-27.3
% torn* (n), 95%CI	13.3 (38), 9.8-17.8	16.9 (41), 12.7-22.1	11.7 (40), 8.7-15.6	28.6 (70), 23.2-34.6
**24 months post intervention**				
Net inspected	303	282	284	188
% good condition (n), 95%CI	44.9 (136), 39.3-50.5	36.5 (103), 31.1-42.3	36.3 (103), 30.9-42.0	33.0 (62), 26.6-40.1
% acceptable condition (n), 95%CI	26.7 (81), 22.0-32.0	24.8 (70), 20.1-30.2	29.9 (85), 24.9-35.5	23.9 (45), 18.3-30.6
% torn* (n), 95%CI	28.4 (86), 23.6-33.7	38.7 (109), 33.1-44.5	33.8 (96), 28.5-39.5	43.1 (81), 36.1-50.3

*Net conditions categorised[1] as good when hole area =<79 cm^2, acceptable = 80-789 cm^2, and torn>=790 cm^2

1.*WHO. Vector Control Technical Expert Group Report to MPAC September 2013: Estimating functional survival of long-lasting insecticidal nets from field data. Geneva, Switzerland: World Health Organization, 2013.*

S5: Concentration of active ingredient (insecticide and PBO synergist)

The table shows the concentration of active ingredient (insecticide and PBO synergist) in the study nets when new and after 12 and 24 months of use in the community.

**Net type**	**Fibre**	**Active ingredient**	**Concentration g/kg**			
			**Specification g/kg (+/-25%)**	**New net (t0) mean (Sd)**	**12 months (t12) mean (Sd)**	**24 months (t24) mean (Sd)**
Standard pyrethroid LLIN	Polyester*	Alpha cypermethrin	5.0 (3.75-6.25)	4.7 (0.4)	2.3 (1.3)	1.6 (1.2)
Pyriproxyfen LLIN	Polyethylene**	Alpha cypermethrin	5.5 (4.125-6.875)	5.3 (0.2)	4.3 (0.8)	3.0 (1.5)
		Pyriproxyfen	5.5 (4.125-6.875)	5.4 (0.2)	2.3 (0.8)	1.5 (0.7)
Chlorfenapyr LLIN	Polyester*	Alpha cypermethrin	2.4 (1.8-3.0)	2.5 (0.3)	1.3 (0.5)	0.7 (0.4)
		Chlorfenapyr	4.8 (3.6-6.0)	5.0 (0.6)	1.4 (1.1)	0.9 (0.7)
PBO LLIN	Polyethylene**	permethrin	20 (15-25)	19.4 (0.4)	12.9 (3.2)	10.5 (3.3)
		PBO	10 (7.5-12.5)	9.6 (0.3)	4.0 (1.4)	2.9 (1.7)

*Polyester nets coated with the AI plus a binder to make them wash resistance

** AI and PBO incorporated into polyethylene fibres.

S6: Reduction in pyrethroid and second AI content over time

The figures show the reduction in pyrethroid and second AI content compared to initial content (t0) in each of the study nets after 12 months (t12) and after 24 months (t24).

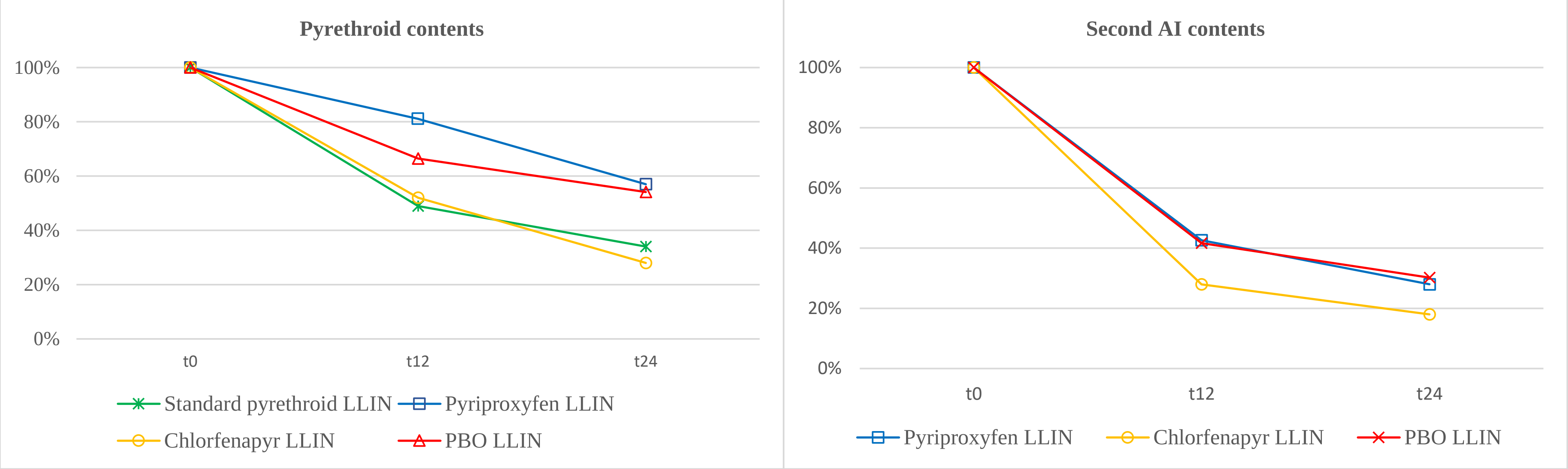

III. Epidemiology.

S7: Consent during malaria prevalence cross-sectional surveys

	Standard pyrethroid LLIN arm	Pyriproxyfen LLIN arm	Chlorfenapyr LLIN arm	PBO LLIN arm
**12 months post intervention**				
Household selected	945	945	945	945
Consent given	643 (68.0%)	619 (65.5%)	662 (70.1%)	616 (65.2%)
No children 6 months to 15 years	142 (15.0%)	151 (16.0%)	113 (12.0%)	161 (17.0%)
Refused	23 (2.4%)	12 (1.3%)	22 (2.3%)	23 (2.4%)
Households (HH) vacant for survey duration	85 (9.0%)	88 (9.3%)	90 (9.5%)	95 (10.1%)
HH not found	52 (5.5%)	71 (7.5%)	57 (6.0%)	50 (5.3%)
HH not visited	0	4 (0.4%)	1 (0.1%)	0
**18 months post intervention**				
Houses selected	945	945	945	945
Consent given	675 (71.4%)	657 (69.5%)	689 (72.9%)	635 (67.2%)
No children 6 months to 15 years	112 (11.9%)	119 (12.6%)	112 (11.9%)	136 (14.4%)
Refused	12 (1.3%)	18 (1.9%)	16 (1.7%)	13 (1.4%)
HH vacant for survey duration	85 (9%)	83 (8.8%)	65 (6.9%)	117 (12.4%)
HH not found	61 (6.5%)	68 (7.2%)	63 (6.7%)	44 (4.7%)
HH not visited	0	0	0	0
**24 months post intervention**				
Houses selected	945	945	945	945
Consent given	662 (70.1%)	689 (72.9%)	693 (73.3%)	692 (73.2)
No children 6 months to 15 years	96 (10.2%)	80 (8.5%)	68 (7.2%)	86 (9.1%)
Refused	14 (1.5%)	13 (1.4%)	17 (1.8%)	16 (1.7%)
HH vacant for survey duration	90 (9.5%)	87 (9.2%)	87 (9.2%)	89 (9.4%)
HH not found	82 (8.7%)	72 (7.6%)	80 (8.5%)	62 (6.6%)
HH not visited	1 (0.1%)	4 (0.4%)	0	0

S8: Side effects from using the study nets

The table shows the proportion of participants in the prevalence cross-sectional surveys reporting side effects from using the net, which are usually associated with pyrethroids.

**Survey: % (n/N)**	Standard pyrethroid LLIN	Pyriproxyfen LLIN	Chlorfenapyr LLIN	PBO LLIN
3 months post intervention	44.1 (90/204)	38.8 (80/206)	8.5 (17/199)	8.5 (17/199)
12 months post intervention	10.1 (167/1647)	9.3 (143/1543)	1.4 (25/1778)	2.0 (31/1539)
18 months post intervention	0.1 (2/1579)	0.0 (0/1425)	0.0 (0/1565)	0.1 (1/1402)
24 months post intervention	0.7 (11/1683)	0.4 (7/1692)	0.2 (3/1744)	0.2 (4/1605)

S9: Type of side effects

The table shows the different types of side-effects reported from using the net as a percentage of all side-effects reported.

	Standard pyrethroid LLIN	Pyriproxyfen LLIN	Chlorfenapyr LLIN	PBO LLIN
**3 months post intervention**				
Skin irritation or paraesthesia	68% (61/90)	64% (51/80)	71% (12/17)	53% (9/17)
Facial burning	28% (25/90)	31% (25/80)	6% (1/17)	41% (7/17)
Runny eyes or nose or sneezing	2% (2/90)	5% (4/80)	0	6% (1/17)
Headache	0	0	12% (2/17)	0
Other	2% (2/90)	0	12% (2/17)	0
**12 months post intervention**				
Skin irritation or paraesthesia	93% (156/167)	97% (139/143)	88% (22/25)	77% (24/31)
Facial burning	4% (6/167)	3% (4/143)	4% (1/25)	0
Runny eyes or nose or sneezing	2% (4/167)	0	8% (2/25)	0
Headache	0	0	0	13% (4/31)
Other	1% (1/167)	0	0	10% (3/31)
**18 month post intervention**				
Skin irritation or paraesthesia	100% (2/2)	0	0	0
Facial burning	0	0	0	0
Runny eyes or nose or sneezing	0	0	0	0
Headache	0	0	0	100% (1/1)
Other	0	0	0	0
**24 month post intervention**				
Skin irritation or paraesthesia	91% (10/11)	100% (7/7)	33% (1/3)	75% (3/4)
Facial burning	9% (1/11)	0	0	0
Runny eyes or nose or sneezing	0	0	33% (1/3)	25% (1/4)
Headache	0	0	0	0
Other	0	0	33% (1/3)	0

S10: Secondary analysis of malaria prevalence: Odds ratio and relative risk

The table presents secondary analyses of malaria prevalence alongside the primary analysis of odds ratios, which is presented in the main paper. To estimate the relative risk (RR), which reflects the relative reduction in malaria prevalence between the standard LLIN arm and each dual AI LLIN intervention arm at each survey timepoint, we used a mixed effects GLM with a log link, Poisson family and robust standard errors; similar results were observed as for other measures of effect, with a RR=0.63(95CI: 0.49 to 0.82, p=0.0005) observed in the CFP-Py LLIN arm at 24 months post-intervention.

Intervention	Survey	Malaria prevalence (%)	OR (95%CI), p-value	RR (95%CI), p-value
Standard pyrethroid LLIN	12 months	31.2%	1	1
	18 months	52.3%	1	1
	24 months	45.8%	1	1
Pyriproxyfen LLIN	12 months	21.7%	0.69 (0.48-1.04), 0.0754	0.78 (0.55 to 1.08), 0.1385
	18 months	50.6%	0.98 (0.67-1.44), 0.9184	1.04 (0.86 to 1.25), 0.6986
	24 months	37.5%	0.79 (0.54-1.17), 0.2354	0.92 (0.69 to 1.23), 0.5831
Chlorfenapyr LLIN	12 months	15.6%	0.47 (0.31-0.71), 0.0003	0.57 (0.41 to 0.77), 0.0004
	18 months	40.9%	0.66 (0.45-0.97), 0.0365	0.85 (0.71 to 1.02), 0.0854
	24 months	25.6%	0.45 (0.30-0.67), 0.0001	0.63 (0.49 to 0.82), 0.0005
PBO LLIN	12 months	19.2%	0.65 (0.44-0.99), 0.0421	0.73 (0.54 to 0.99), 0.0418
	18 months	43.3%	0.76 (0.52-1.12), 0.1699	0.90 (0.74 to 1.09), 0.3003
	24 months	40.7%	0.99 (0.67-1.45), 0.9607	1.02 (0.81 to 1.29), 0.8571

p<0.017 = statistically significant

S11: Incidence sensitivity analysis

This table presents re-analysis of the incidence estimates presented in the main paper, without the period when the cohort follow-up was changed in response to the COVID-19 pandemic.

	Number of clinical episodes	Follow up time child years	Incidence per child per year	Rate ratio	95%CI	p value*
Pyrethroid LLIN						
Year 1	194	605.1	0.32	1		
Year 2	269	785.5	0.34	1		
Overall	463	1390.6	0.33	1		
Pyriproxyfen LLIN						
Year 1	161	604.5	0.27	0.93	0.60-1.46	0.7612
Year 2	267	774.8	0.34	1.10	0.71-1.67	0.6819
Overall	428	1379.3	0.31	1.02	0.68-1.60	0.9076
Chlorfenapyr LLIN						
Year 1	79	603.8	0.13	0.46	0.28-0.74	0.0013
Year 2	142	784.3	0.18	0.58	0.38-0.91	0.0175
Overall	221	1388.1	0.16	0.53	0.35-0.81	0.0032
PBO LLIN						
Year 1	79	591.8	0.13	0.51	0.32-0.81	0.0047
Year 2	270	779.0	0.35	1.25	0.82-1.90	0.2949
Overall	349	1370.9	0.25	0.93	0.62-1.40	0.7333

P value for the time by study arm interaction term is <0.0001. Each intervention arm is compared to the standard LLIN arm for the same time point. Rate ratios are adjusted for baseline cluster-level variables used in restricted randomisation. *A p value <0.017 was considered statistically significant after Bonferroni correction. LLIN=long-lasting insecticidal net, PBO=piperonyl butoxide.

S12: Monthly malaria cases

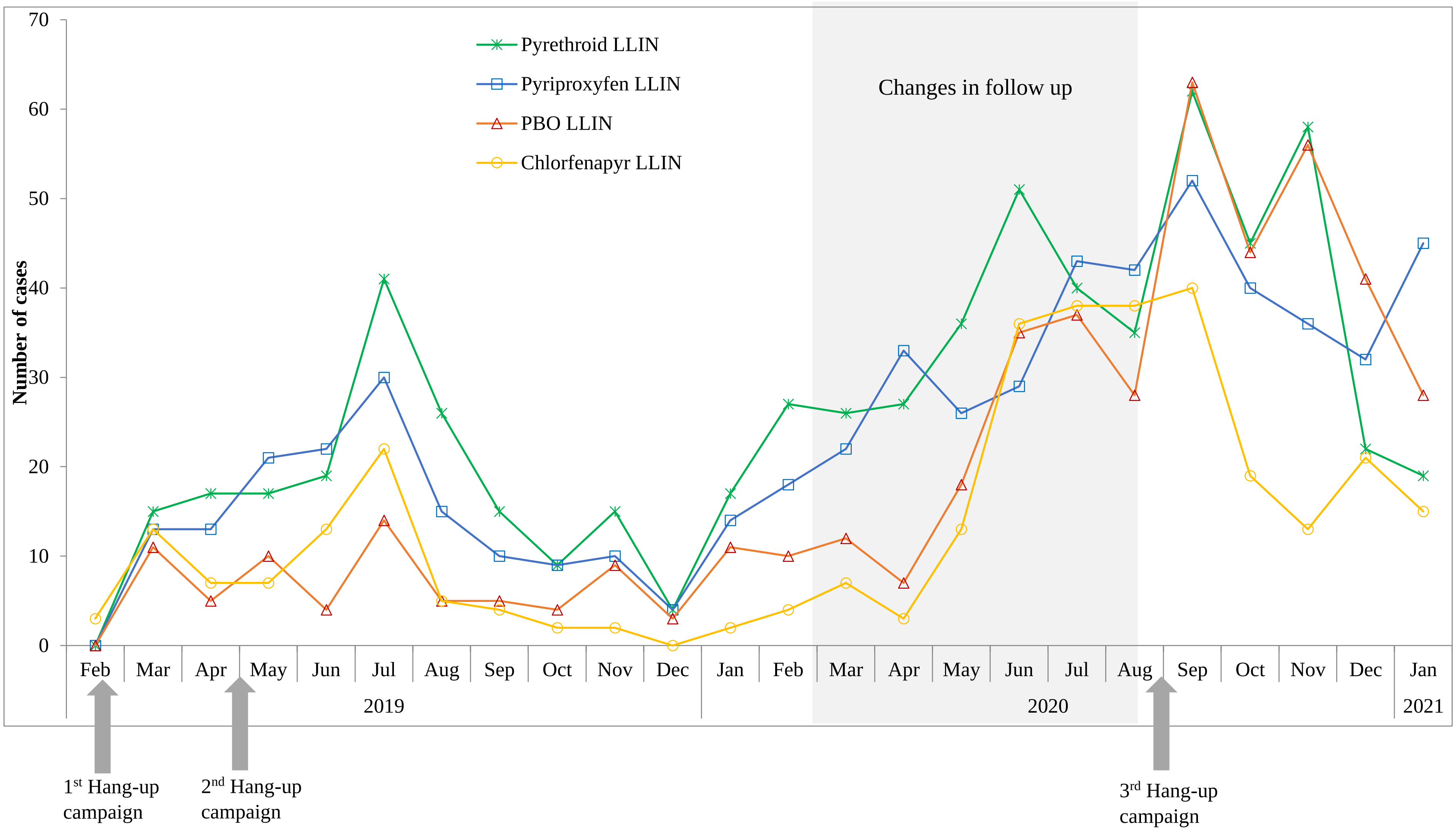

S13: Anaemia prevalence in children from 6 months to 5 years old

	Children with anaemia	Children tested	Prevalence of anaemia	OR	95%CI	p value*
Pyrethroid LLIN arm						
12 months	7	374	1·9%	1		
18 months	45	443	10·2%	1		
24 months	29	399	7·3%	1		
Pyriproxyfen LLIN arm						
12 months	8	350	2·3%	1·15	0·40-3·32	0·794
18 months	37	383	9·7%	0·88	0·52-1·48	0·6308
24 months	38	434	8·8%	1·15	0·66-2·03	0·6176
Chlorfenapyr LLIN arm						
12 months	11	396	2·8%	1·55	0·58-4·19	0·3833
18 months	33	428	7·7%	0·72	0·42-1·23	0·2254
24 months	28	416	6·7%	0·94	0·52-1·70	0·8326
PBO LLIN arm						
12 months	10	358	2·8%	1·62	0·59-4·43	0·3487
18 months	23	382	6·0%	0·58	0·33-1·03	0·0626
24 months	26	400	6·5%	0·93	0·51-1·68	0·8038

P value for the time by study arm interaction term is 0·5728. Each intervention arm is compared to the pyrethroid LLIN arm for the same time point. Odd ratios are adjusted for baseline cluster-level variables used in restricted randomisation. *A p value <0·017 was considered statistically significant after Bonferroni correction. LLIN=long-lasting insecticidal net. PBO=piperonyl butoxide.

IV. Entomology

S14: Consent during entomological data collection

	Standard pyrethroid LLIN	Pyriproxyfen LLIN	Chlorfenapyr LLIN	PBO LLIN
Year 1				
Households (HH) selected	1092	1092	1092	1092
Consent given	670 (61.4%)	672 (61.5%)	671 (61.4%)	672 (61.5%)
HH not found	13 (1.2%)	22 (2.0%)	21 (1.9%)	16 (1.5%)
HH not visited	316 (28.9%)	273 (25.0%)	293 (26.8%)	258 (23.6%)
HH vacant	82 (7.5%)	112 (10.3%)	96 (8.8%)	127 (11.6%)
Refused	11 (1.0%)	13 (1.2%)	11 (1.1%)	19 (1.7%)
Year 2				
Households (HH) selected	1365	1365	1365	1365
Consent given	838 (61.4%)	840 (61.5%)	840 (61.5%)	840 (61.5%)
HH not found	36 (2.6%)	34 (2.5%)	56 (4.1%)	34 (2.5%)
HH not visited	302 (22.1%)	305 (22.3%)	303 (22.2%)	298 (21.8%)
HH vacant	162 (11.9%)	175 (12.8%)	153 (11.2%)	170 (12.5%)
Refused	27 (2.0)	11 (0.8%)	13 (1.0%)	23 (1.7%)

¶

S15. Insecticide resistance results

Table S13 presents the average 30 min mortality (diagnostic time for the CDC bottle bioassay), following insecticide exposure of wild populations of *An. funestus* s.l. and *An. gambiae* s.l.

For *An. funestus* s.l. average 72-hr mortality (diagnostic time for CFP) across 4 study clusters (#63, 72, 73 and 78) was 99.8% at the tentative diagnostic dose of CFP (100μg/ml). For *An. gambiae* s.l. average 72-hr mortality across 2 study clusters (#63, 68) was 95.1% at the tentative diagnostic dose of CFP.

Complete infertility was observed with the susceptible colony strain *An. gambiae* s.s. Kisumu following exposure to the tentative diagnostic dose of PPF (100μg/ml) and ovarian dissection 3 days later (n=74); however, reduced infertility was observed among *An. funestus* s.l. from study clusters #72, 73 and 78 (average proportion of infertile individuals of 23.7%; n=536) and *An. gambiae* s.l. from study clusters #68 and 72 (average proportion of infertile individuals of 20.5%; n=112).

S16: *Anopheles* mortality 30 minutes after exposure to different doses of insecticides

		Resistance test on *An. funestus*		Resistance test on *An. gambiae*	
**Insecticide**	**Insecticide Dose (X)**	cluster #	**%** *An. funestus* s.l.<br>30-min mortality [95% CI; n tested]	cluster #	*% An. gambiae* s.l.<br>30-min mortality [95% CI; n tested]
Alpha-cypermethrin	1	31, 63, 68, 72, 73, 78	32.5% [29.4-35.8%; 809]	41, 63	33.3% [27.6-39.6%; 237]
	2	31, 63, 68, 72, 73, 78	50.0% [46.5-53.5%; 766]	41, 63	43.9% [36.7-51.5%; 173]
	5	31, 63, 68, 72, 73, 78	56.0% [52.5-59.5%; 768]	41, 63	93.6% [86.4-97.1%; 94]
	10	31, 63, 68, 72, 73, 78	71.7% [68.2-74.9%; 702]	41	100% [40]
Permethrin	1	31, 63, 68, 72, 73	42.2% [38.7-46.1%; 589]	68	46.0% [37.0%-55.3%; 113]
	2	31, 63, 68, 72, 73	55.3% [51.2-59.4%; 553]	68	65.2% [55.8-73.5%; 112]
	5	31, 63, 68, 72, 73	69.0% [64.9-72.7%; 538]	68	93.2% [86.8-96%; 117]
	10	31, 63, 72, 73	84.8% [81.4-87.8%; 488]	68	97.3% [91.9-99.14%; 111]
PBO + Permethrin	1	63, 72, 73	80.2% [75.9-83.9%; 379]	68	93.4% [88.4-96.3%; 166]
	2	63, 72, 73	84.3% [80.3-87.6%; 386]	68	100% [163]
	5	72, 73	96.3% [93.4-97.9%; 294]	-	-

V. Economic evaluation

S17. Decision tree

The decision tree presents the structure of the model for children aged 6 months to 10 years. The square node indicates the policy decision regarding the choice of nets to distribute. Circular probability nodes show disease progression. Triangular terminal nodes reflect the final outcomes, including costs and disability-adjusted life-years (DALYs).

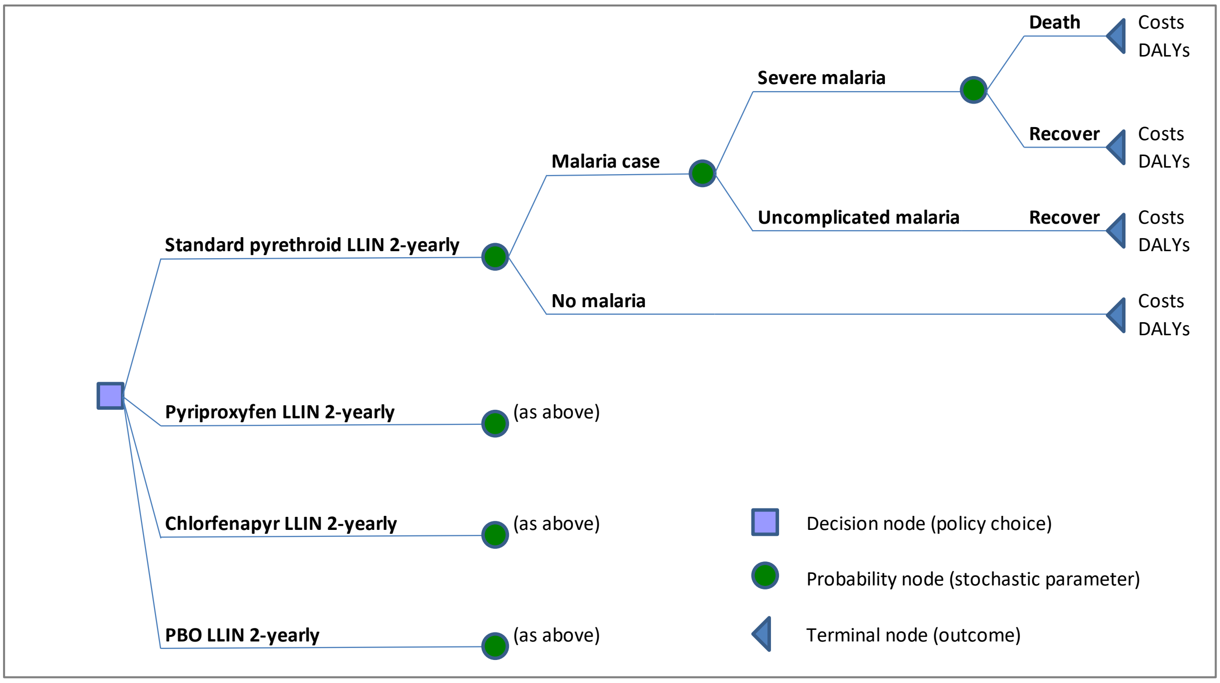

S18. Model structure

This figure shows the model structure for the full (all ages) population. The lower half of the figure shows children aged 6 months to 10 years (in turquoise). The upper half of the figure shows persons aged above 10 years (in purple). Straight turquoise and purple arrows indicate the flow of the population through the model. Curved blue arrows indicate where the population, number of malaria cases, and deaths in people aged over 10 years are estimated as a function of the corresponding number of children aged 6 months to 10 years, number of malaria cases in children, and number of deaths in children (see S20 and S21 for more information). Red ovals indicate health states (severe malaria cases, uncomplicated malaria cases, deaths) at which costs ($) or disability-adjusted life-years (DALYs) are incurred. DALYs: Disability-adjusted life-years; GBD: Global Burden of Disease; WPP: World Population Prospects; $: Costs.

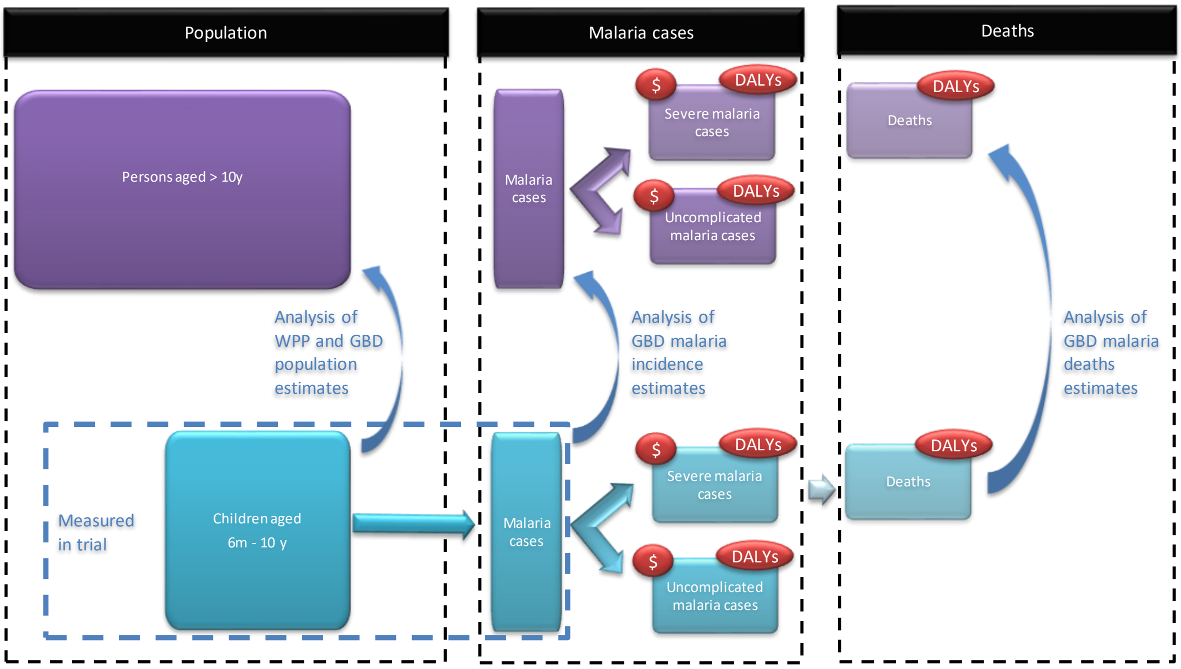

S19. Input parameters for the cost-effectiveness analysis

CU10: children aged 6 months to 10 years; DHS: Demographic and Health Survey; DSA: Deterministic sensitivity analysis; FY: Fiscal year; GBD: Global Burden of Disease; GDP: Gross domestic product; iDSI: International Decision Support Initiative; LMICs: Low- and middle-income countries; PMI: President's Malaria Initative; RDT: Rapid diagnostic test; s.d.: standard deviation; WHO: World Health Organization; WHO-CHOICE: WHO's Choosing Interventions that are Cost-Effective programme. *For beta distributions, a mean and standard deviation are given to facilitate interpretation; the alpha and beta parameters defining the beta distribution are calculated using method of moments from the mean and standard deviation given. (1)

		Probabilistic		Deterministic		
**MODEL ASSUMPTIONS**	**Base case (Mean)**	**Distribution**	**s.d.**	**Low**	**High**	**Justification / Source**
Population of modelled cohort	10,000	NA	NA	NA	NA	Assumption
% of population that is aged 6m - 10y ("CU10")	29.00	NA	NA	NA	NA	World Population Prospects (for 2020)(2) and GBD for 2019(3); Infants <6m estimated as half the population of children <1y; Both sources produced same value.
Discount rate (%)	3.00	NA	NA	1.00	7.00	iDSI Reference Case for Economic Evaluation in LMICs(4)
Maximum incremental cost per DALY averted to be considered cost-effective (cost-effectiveness threshold from health service perspective)	NA	NA	NA	292	393	Highest and lowest of the four fractions of per capita GDP estimated in Ochalek (2018)(5), multiplied by Tanzania's per capita GDP in the most recent year available (2020) from World Bank.(6)
**EFFECTS**						
**Malaria incidence**						
Incidence rate Y1 in CU10 with standard net per 1000 child-years	LN(320.61)	Lognormal	0.216	LN(320.61)	LN(565.92)	Trial estimates based on child cohort – See Table 3 in main paper; Standard deviations are calculated based on the natural logarithm (LN) of the bounds of the 95% confidence intervals calculated from the mean and sample size for the estimate in Table 3.
Incidence rate Y2 in CU10 with standard net per 1000 child-years	LN(565.92)	Lognormal	0.161			
Rate ratio - clinical malaria in CU10 - PBO (Olyset Plus) : Standard (Y1)	LN(0.53)	Lognormal	0.241	NA	NA	Trial estimates based on child cohort – see S8; Standard deviations given reflect the s.d. within the lognormal distribution and are calculated based on the natural logarithm (LN) of the bounds of the 95% confidence intervals presented in S8.
Rate ratio - clinical malaria in CU10 - Pyriproxyfen (Royal Guard) : Standard (Y1)	LN(0.94)	Lognormal	0.230			
Rate ratio - clinical malaria in CU10 - Chlorfenapyr (Interceptor G2) : Standard (Y1)	LN(0.46)	Lognormal	0.248			
Rate ratio - clinical malaria in CU10 - PBO (Olyset Plus) : Standard (Y2)	LN(1.11)	Lognormal	0.211			
Rate ratio - clinical malaria in CU10 - Pyriproxyfen (Royal Guard) : Standard (Y2)	LN(1.02)	Lognormal	0.214			
Rate ratio - clinical malaria in CU10 - Chlorfenapyr (Interceptor G2): Standard (Y2)	LN(0.61)	Lognormal	0.218			

	Base case (Mean)	Probabilistic		Deterministic		Justification / Source
		Distribution	s.d.	Low	High	
% of total malaria cases occurring in CU10 - Standard (Y1)	52.45	Beta*	7.000	NA	1.00	Base case: A function of rate of malaria incidence in CU10 based on analysis of GBD estimates(7) for all countries for 2010-19: y = 0.087ln(x) + 0.0225; s.d. is an assumption, and the alpha and beta parameters for the beta distribution for each of the 8 different parameters are calculated using method of moments based on the base case (mean) and s.d. shown. High: Assumes no cases in persons aged > 10y. See S20 and S21 for further information.
% of total malaria cases occurring in CU10 - PBO (Olyset Plus) (Y1)	46.93	Beta*	7.000			
% of total malaria cases occurring in CU10 - Pyriproxyfen (Royal Guard) (Y1)	51.91	Beta*	7.000			
% of total malaria cases occurring in CU10 - Chlorfenapyr (Interceptor G2) (Y1)	45.70	Beta*	7.000			
% of total malaria cases occurring in CU10 - Standard (Y2)	57.39	Beta*	7.000			
% of total malaria cases occurring in CU10 - PBO (Olyset Plus) (Y2)	58.30	Beta*	7.000			
% of total malaria cases occurring in CU10 - Pyriproxyfen (Royal Guard) (Y2)	57.57	Beta*	7.000			
% of total malaria cases occurring in CU10 – Chlorfenapyr (Interceptor G2) (Y2)	53.09	Beta*	7.000			
**Disability-adjusted life-years (DALYs)**						
% cases in CU10 that would become severe	4.00	Beta*	0.800	NA	NA	Calibrated to ensure modelled case fatality rates: 1) lie between all-age estimates for Tanzania by WHO (0.31% all ages)(8) and GBD (0.25% all ages)(7); 2) are consistent with GBD case fatality rates for CU10 (0.41%)(7) and for persons >10y (0.09%)(7); and 3) are consistent with PMI Tanzania Operational Plan FY 2021 estimates of the proportion of all cases that would be severe in 2021 (4%).(9) s.d. set at 20% of base value.
% severe cases in CU10 that result would in death	10.00	Beta*	2.000	NA	NA	
% cases in persons > 10 years that would become severe	2.00	Beta*	0.400	NA	NA	
% severe cases in persons > 10 years that would result in death	10.00	Beta*	2.000	NA	NA	
% of total malaria deaths occurring in CU10	86.00	Beta*	7.000	NA	1.00	Base case: Based on analysis of GBD data for Tanzania (in the context of all countries) for 2010-19(7); High: Assumes no deaths in persons aged > 10y.
Years of life lost per death in CU10 (discounted)	29.03	NA	NA	NA	NA	WHO life tables for Tanzania for 2019,(10) disaggregated into 5-year age groups; values reflect means of the discounted years of life lost for a death in each age group, weighted by the number of malaria deaths in Tanzania by 5-year age group estimated by GBD.(7)
Years of life lost per death in persons > 10 years (discounted)	21.76	NA	NA	NA	NA	
Duration of uncomplicated malaria (days)	3.00	Gamma	0.001	NA	NA	Assumption
Duration of severe malaria (days)	7.00	Gamma	0.002	NA	NA	
DALY weight - uncomplicated malaria (infectious disease moderate)	0.05	Beta*	0.011	NA	NA	Salomon et al, 2015(11)
DALY weight - severe malaria (infectious disease severe)	0.13	Beta*	0.026	NA	NA	

COSTS (constant 2020 United States dollars)	Base case (Mean)	Probabilistic Distribution	Probabilistic s.d.	Deterministic Low	Deterministic High	Justification / Source
**Costs of nets**						
Cost of net procurement per net – Standard	$2.07	NA	NA	NA	NA	Global Fund pooled procurement mechanism reference price list,(12) based on 180cm x 160cm x 180cm white net with standard accessories (6 hooks and strings, bag, normal inserts). Deterministic value for PBO reflect values used in threshold analysis, showing value at which the strategy would lie on the lower cost-effectiveness threshold; this is not necessarily considered a plausible value.
Cost of net procurement per net – PBO	$2.98	NA	NA	NA	$3.72	
Cost of net procurement per net – Pyriproxyfen (Royal Guard)	$3.68	NA	NA	$1.68	NA	Base case: Correspondence with the Global Fund based on 180cm x 160cm x 180cm white net with standard accessories (6 hooks and strings, bag, normal inserts). Deterministic values reflect values used in threshold analysis (S25b), showing value at which the strategy would lie on the lower cost-effectiveness threshold; they are not necessarily considered plausible values.
Cost of net procurement per net - Chlorfenapyr (Interceptor G2)	$3.02	NA	NA	NA	$10.13	
Number of people per net distributed	1.70	NA	NA	NA	NA	Trial administrative records
Cost of net distribution per net	NA	NA	NA	NA	NA	Not applicable in this analysis because distribution costs are assumed identical across the comparators.
**Costs of illness and case management**						
% Seeking care for uncomplicated	79.00	Beta*	10.000	NA	NA	Tanzania's DHS 2015-16(13) indicated that care was sought for 80% of children under 5y with fever in the last 2w; this estimate was assumed to apply to all ages. The proportion seeking care for uncomplicated malaria was estimated by assuming that care is sought for 95% of people with severe malaria. These values are lower than operational planning, which assumes 95% of fever cases will receive RDT.
% Seeking care for severe	95.00	Beta*	10.000	NA	NA	
% of care sought that is publicly (not privately) financed	60.00	Beta*	10.000	30.000	90.000	PMI Tanzania Operational Plan FY 2021(9) indicates that 60% of care is sought in private facilities. Some people seeking care in private facilities are able to be reimbursed by health insurance schemes, which are publicly subsidized, making the public provider the ultimate payer. Some people seeking care in public facilities are required to pay user fees; however, there are exemptions for pregnant women, children, the elderly, and the poorest. With limited local data on this complex and rapidly-changing health financing landscape, a simplifying assumption was made and fairly extreme range for the deterministic sensitivity analyses was used.
% of care-seekers with uncomplicated malaria receiving RDT+ACT	95.00	Beta*	10.000	NA	NA	PMI Tanzania Operational Plan FY 2021(9) calculates RDT needs as 95% of all malaria cases.

	Base case (Mean)	Probabilistic		Deterministic		Justification / Source
		Distribution	s.d.	Low	High	
% of care-seekers with severe malaria receiving RDT+ACT	95.00	NA	NA	NA	NA	Assumption
Cost per case diagnosed and treated	Varies with perspective, severity, and facility type	Gamma	Varies	NA	NA	Base case calculated as sum of components relevant to perspective taken, separately for uncomplicated and severe malaria; s.d. is assumed 20% of base case.
Cost of rapid diagnostic test (Antigen Pf / Pan, no accessories) (to donor)	$0.33	NA	NA	NA	NA	Global Fund pooled procurement mechanism reference pricing: RDTs (22 July 2020)(14)
Cost of medicines (AL) for uncomplicated (to donor in public facilities, to household in private facilities)	$0.51	NA	NA	NA	NA	Global Fund pooled procurement mechanism reference pricing: Antimalaria medicines (Q1 2021)(15); Mean of prices of different doses, weighted by age structure of malaria cases using GBD incidence estimates for Tanzania for 2019.(7) Artesunate costed assuming 6 vials per dose based on PMI Tanzania Operational Plan FY 2021.(9)
Cost of medicines (AL+injectable artesunate) for severe case (to donor)	$13.19	NA	NA	NA	NA	
Cost of freight and insurance (as % of cost of RDTs and medicines)	10.00	NA	NA	NA	NA	Assumption
Cost per consultation in public facility (to government)	$0.74	NA	NA	NA	NA	WHO-CHOICE Unit Cost Estimates for Service Delivery(16): rural health facility with no beds in Tanzania; inflated based on Tanzania's GDP and converted to USD based on 2020 exchange rates. Assumes consultations are provided free of charge in public facilities and patients pay for consultation in private facilities.
Cost per consultation in private facility (to household)	$1.04	NA	NA	NA	NA	
Cost of hospitalisation per day in public facility (to government)	$3.79	NA	NA	NA	NA	WHO-CHOICE Unit Cost Estimates for Service Delivery(16): rural, public, primary-level hospital in Tanzania
Duration of hospitalisation - severe (days)	5.000	NA	NA	NA	NA	Assumption
Cost of time spent ill per day (to household)	$1.54	NA	NA	NA	NA	50% of per capita GDP per day for Tanzania (World Bank)(6)
Exchange rate: Tanzania shillings per United States dollar (2020)	TZS 2,294	NA	NA	NA	NA	World Development Indicators(6)

S20. Modelling of cases and deaths in persons > 10 years

In the trial, malaria incidence was only measured in children aged 6 months to 10 years (CU10); however, LLINs may reduce malaria incidence and associated mortality in people of all ages. To capture the full health benefits of more effective LLINs, incidence and deaths in persons >10 years were modelled. In doing so, we sought to account for the epidemiology of malaria – specifically, that at higher population-level malaria incidence rates, more of the overall incidence is concentrated amongst younger age groups.

We therefore analysed modelled estimates from the Global Burden of Disease (GBD) study to understand the relationship between malaria incidence in CU10 (as estimated in the trial) and the proportion of all malaria cases that are in CU10. Similarly, we used the GBD to estimate the relationship between malaria death rates in CU10 (as modelled from the trial data) and the proportion of all malaria deaths that are in CU10. We created two datasets in which each data point reflected estimates for a single country-year. All countries in the GBD were included with data points for each of the most recent 10 years available (2010-19). These data are presented in blue in Figure S21, with the ten data points for Tanzania presented in red.

According to GBD estimates, in Tanzania in 2010, 55% of all malaria cases were in children under 10, and there were 243 cases per 1,000 children under 10. By 2019, the proportion of cases had fallen to 50%, while incidence fell to 198 cases per 1,000. These incidence rates lay between, but fairly distant from the upper and lower incidence bounds observed in the trial of 130 cases per 1,000 (in the chlorfenapyr LLIN arm in Y1) and 570 cases per 1,000 (in the standard arm in Y2). A log-linear regression was therefore conducted on all country-years of data (Figure S21), which produced a flatter, less extreme relationship than the Tanzania data points alone, and avoided extrapolation far beyond the range of the Tanzania data points while remaining broadly consistent with them. The estimates of the proportion of all cases expected to occur in children under 10 at the incidence rates observed in the four trial arms and 2 trial years are presented in Table S19.

Also according to GBD estimates, in Tanzania in 2010, 87% of all malaria deaths were in children under 10, and there were 122 malaria deaths per 100,000 children under 10. This malaria death rate was close to, but slightly lower than, the lowest death rate modelled for children under 10 in the trial (129 malaria deaths per 100,000 children under 10 with chlorfenapyr in Y1), and far lower than the highest mean death rate modelled (427 deaths per 100,000 children under 10 with standard LLINs in Y2). After 2010, GBD estimates indicated that Tanzania's malaria death rate fell rapidly – to 80 cases per 100,000 in 2012 – but the proportion of deaths that were in children remained as high as 86%. Compared with other countries, these proportions were near the top of the range; only Ethiopia had a higher proportion, with 93% of deaths in 2010 occurring in children under 10, falling to 89% of deaths in 2019. Given that the data for all countries were suggestive of a plateau, for the cost-effectiveness analysis, we modelled the proportion of deaths that were in children as a fixed proportion, with mean of 86%, regardless of the malaria mortality rate.

S21. Relationship between age and malaria incidence and death rates

The figures present estimates from the Global Burden of Disease (GBD) for the years 2010-19, with our analysis and values used in the cost-effectiveness analysis superimposed. Each blue point represents a single country-year of GBD data for a country with non-zero malaria incidence (upper figure) or non-zero deaths (lower figure). Each red point represents a country-year of GBD data specific to Tanzania. Orange vertical lines represent the lowest and highest of the mean values for the four strategies compared in the trial in each of the two years for the incidence (upper figure) and death rate (lower figure). Dotted turquoise lines show the equation used to calculate the proportion of all cases and deaths that would be expected to have occurred in children aged 6 months to 10 years as a function of the incidence rate or death rate in that age group.

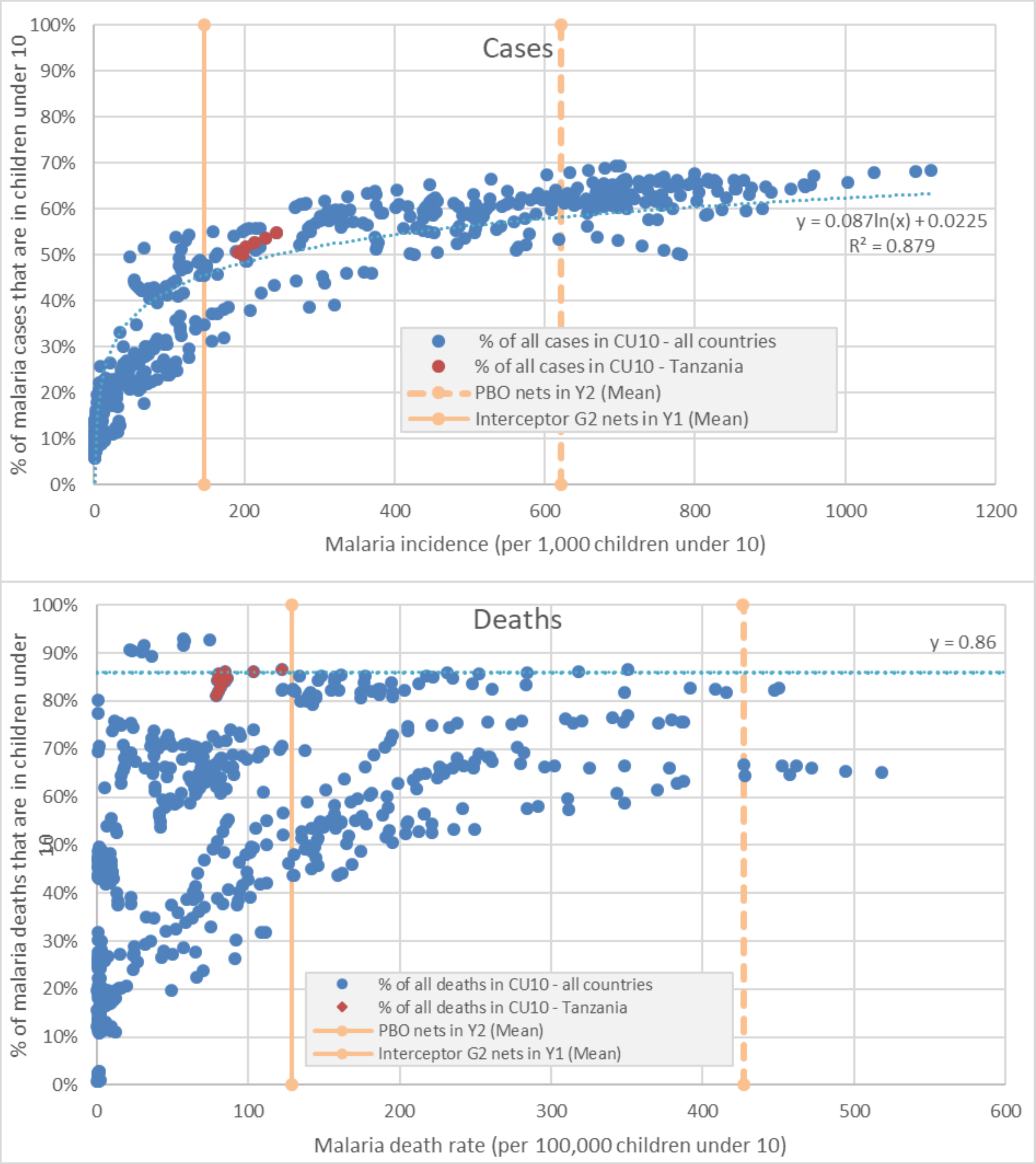

S22. Calculation of disability-adjusted life-years (DALYs)

Malaria DALYs under each strategy were estimated as the sum of years of life lived with disability (YLDs) and years of life lost due to premature mortality (YLLs) with no age weighting and a 3% annual discount rate (in the base case). YLDs for all malaria cases were calculated separately for uncomplicated (requiring outpatient treatment only) and severe cases (defined as requiring hospitalisation) as the product of the population size, the incidence each year with standard nets, the rate ratio for that year, the probability of a case becoming severe, the duration of a case, and the disability weight for infectious disease (moderate or severe).(11)

YLLs were estimated using WHO life tables for Tanzania.(17) Discounting was applied to remaining life expectancy for each 5-year age cohort. Mean discounted remaining life expectancy was then calculated for each of the two age cohorts in the model (CU10 vs. persons > 10y), weighted by the number of malaria deaths expected in each 5-year age cohort according to GBD estimates for Tanzania. Mean YLLs per death for each of the two age cohorts were then combined with modelled estimates of the number of deaths in each age cohort to estimate total YLLs for each strategy.

S23. Cases, deaths, DALYs, and costs by net type, year, and age group

The following four panels disaggregate health effects and costs based on the deterministic cost-effectiveness analysis of the simulated cohort of 10,000 people. All values are shown without discounting. The cases, deaths, and DALYs figures illustrate the share of the total accounted for by children aged 6m to 10y, for which estimates are based directly on malaria case incidence measured in the trial. Cases, deaths, and DALYs in persons older than 10 years are estimated as a function of incidence and deaths in children under 10 using outputs from Global Burden of Disease model outputs (see Text S2, Figure S6). Costs are presented in constant 2020 United States dollars. CU10: Children aged 6m to 10y; DALYs: Disability-adjusted life-years; Dx: Diagnosis; LLINs: Long-lasting insecticide-treated bed nets (purchase price only); Tx: Treatment; YLD: Years of life lived with disability; YLL: Years of life lost to premature mortality.

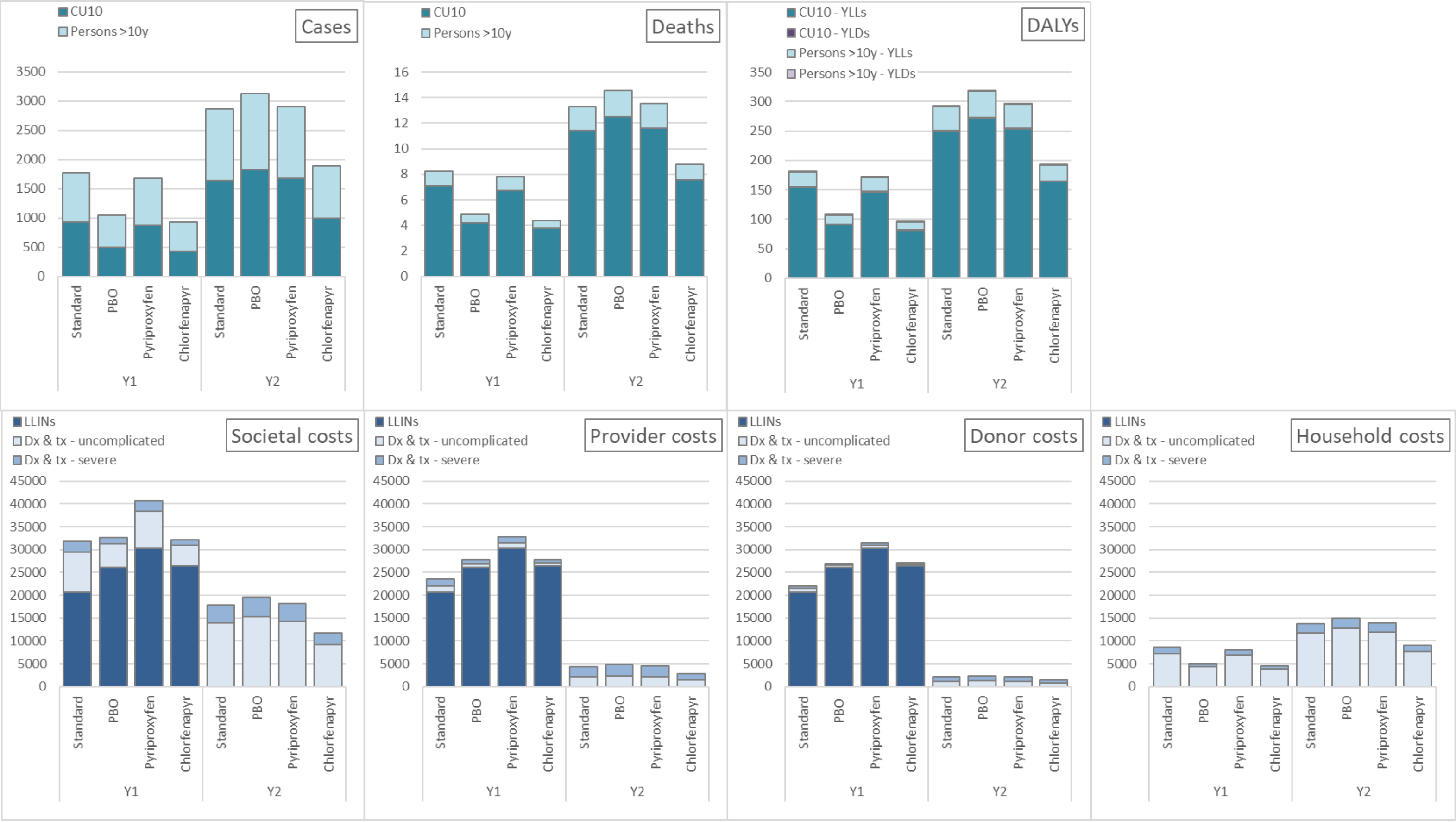

S24. Mean costs, effects, and incremental cost-effectiveness ratios

The tables below present the mean costs and effects of each of the four nets (S24a), and the incremental costs and effects (S24b) and cost-effectiveness (S24c) of the three dual active ingredient nets compared to standard nets over the 2-year period of the trial. Results presented reflect means of probabilistic uncertainty analysis and correspond with mean values presented on cost-effectiveness planes (Figure 3 in main paper) for the modelled cohort of 10,000 people (all ages). The combined costs of net procurement and diagnosis and treatment of malaria cases are presented for the societal, public provider, and donor perspectives; costs of net distribution are not included as they would be identical across strategies. The public provider perspective includes both costs to donors and costs to the domestically-funded public health service. The societal perspective includes both costs to public providers and also to households. For each dual active ingredient net, if it is both more costly and more effective than standard nets, the incremental cost per DALY averted compared with standard nets is shown. If the dual active ingredient net is less costly and more effective, it is described as "dominant"; if the dual AI net is more costly and less effective, it is described as "dominated"; in the case of dominant or dominated strategies, no incremental cost-effectiveness ratio (ICER) is shown because the ICER would be negative and difficult to interpret.

S24a. Mean costs and effects

	Costs (constant 2020 USD)				Effects	
LLIN type	Societal	Household	Public provider	Donor	Cases	DALYs
Standard 2-yearly	50,329	22,486	28,040	24,281	4,785	412
PBO 2-yearly	53,007	20,569	32,856	29,333	4,378	375
Pyriproxyfen 2-yearly	60,410	22,808	37,661	33,810	4,886	422
Chlorfenapyr 2-yearly	45,016	14,087	30,934	28,585	3,026	260

S24b. Incremental costs and effects with respect to standard nets

	Incremental costs (constant 2020 USD, standard deviation)				Incremental effects (standard deviation)	
LLIN type	Societal	Household	Public provider	Donor	Cases	DALYs
Standard 2-yearly	Reference				Reference	
PBO 2-yearly	2,677 (5,051)	-1,916 (3,736)	4,816 (1,360)	5,053 (572)	-408 (845)	-37 (72)
Pyriproxyfen 2-yearly	10,081 (4,950)	323 (3,866)	9,621 (1,327)	9,529 (588)	100 (825)	9 (71)
Chlorfenapyr 2-yearly	-5,313 (4,415)	-8,398 (3,493)	2,894 (1,129)	4,304 (505)	-1,759 (834)	-152 (72)

S24c. Incremental cost-effectiveness ratios with respect to standard nets

	Incremental USD per DALY averted (uncertainty interval)			
LLIN type	Societal	Household	Public provider	Donor
Standard 2-yearly	Reference			
PBO 2-yearly	72 (dominant, dominated)	Dominant (dominant, dominated)	130 (12, dominated)	136 (22, dominated)
Pyriproxyfen 2-yearly	Dominated (14, dominated)	Dominant (dominant, dominated)	Dominated (61, dominated)	Dominated (73, dominated)
Chlorfenapyr 2-yearly	Dominant (dominant, dominant)	Dominant (dominant, dominant)	19 (1, 105)	28 (11, 120)

S25. Economic evaluation sensitivity analysis results

To explore the influence of uncertainty and heterogeneity in individual parameters on cost-effectiveness results, the probabilistic uncertainty analyses presented in the main paper (Figure 2) were re-run, with the value of one key parameter at a time held constant at a value different from the base case (Table S19). In this way, the analyses remain probabilistic, and continue to show the combined uncertainty in results, while also showing how overall results would vary if one key parameter were held constant at a value higher or lower than its mean in the base case. While various methods have been proposed to combine deterministic and probabilistic sensitivity analyses in similar ways, they are difficult to adapt to multi-way comparisons such as this one, which is why this particular approach was taken. These sensitivity analyses are all presented from the (public) provider perspective.

The bowtie shape of the distribution of results on the cost-effectiveness plane for pyriproxyfen and PBO nets occurs because the rate ratios for the effectiveness of each of these nets with respect to standard nets includes 1 and the cost of the nets themselves has been modelled as a deterministic parameter. For a given iteration, if the random draw for the rate ratio is 1, there are no incremental effects relative to standard nets (and so the point is on y-axis), and the incremental costs relative to standard nets only consist of the additional cost of the nets themselves (a fixed parameter).

S25a shows the impact of plausible variation in all key individual parameters except for the price of nets. S25b shows the variation in the price of each of the dual AI nets that would be necessary to change the recommendation about whether switching from standard nets to that specific dual AI net would be cost-effective at the more conservative cost-effectiveness threshold.

CE: Cost-effectiveness; USD: United States dollars.

S25a. Impact of plausible variation in individual variables on cost-effectiveness

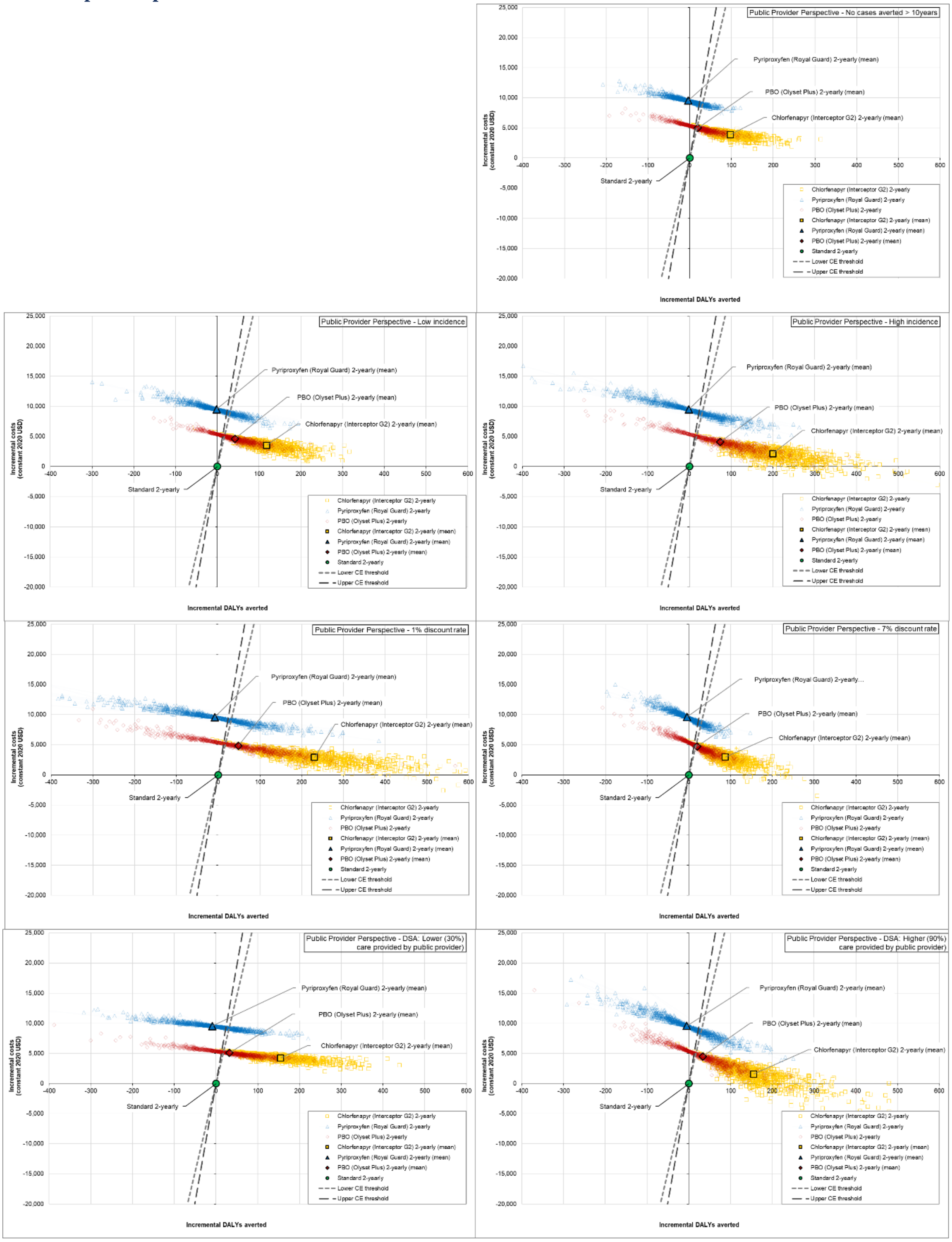

S25b. Threshold analysis: At what price of each dual AI net would cost-effectiveness conclusions change?
This figure shows a threshold analysis, which identifies the price of each of the dual AI nets at which the incremental cost-effectiveness ratio would equal the more conservative (lower) cost-effectiveness threshold with respect to standard nets, and therefore the point at which the determination of cost-effectiveness would change. Assuming the cost of standard pyrethroid-only nets remains constant, PBO and chlorfenapyr LLINs would remain cost-effective relative to the more conservative cost-effectiveness threshold if their prices increased up to $3.72 and $10.13 per net, respectively, and Pyriproxyfen LLINs would become cost-effective if their price fell to $1.68 per net (appendix, p. 30). If the cost of standard pyrethroid-only nets were to change, the threshold prices shown here would change by the same absolute value; for example, if the cost of standard nets were to increase to $3.07, then chlorfenapyr LLINs would remain cost-effective up to a price of $11.13 in this context, assuming all else remained equal.

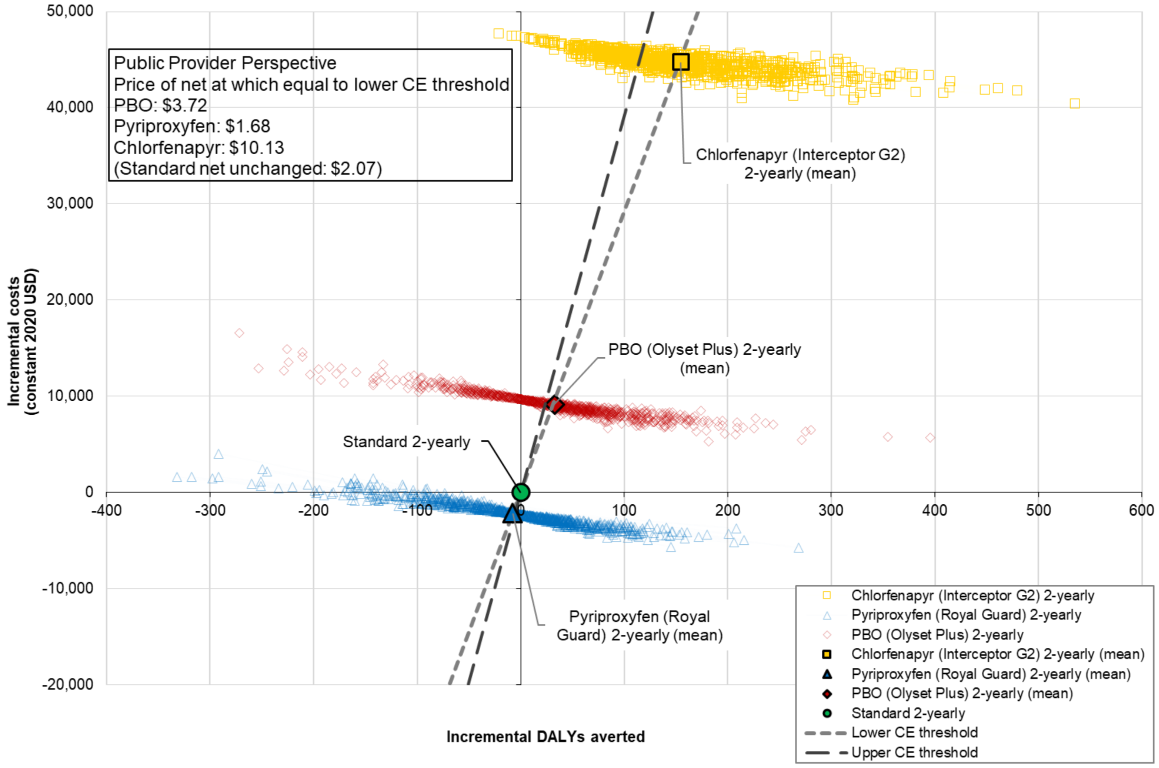

VI. References

1. Briggs AH, Claxton K, Sculpher MJ. Decision Modelling for Health Economic Evaluation: Oxford University Press; 2006.
2. Population Division of the Department of Economic and Social Affairs. World Population Prospects 2019, custom data acquired via website. In: Nations U, editor. 2019.
3. Global Burden of Disease Collaborative Network. Global Burden of Disease Study 2019 (GBD 2019) Population Estimates 1950-2019 Seattle, United States of America: Institute for Health Metrics and Evaluation (IHME); 2020 [updated 29 January 2021. Available from: http://ghdx.healthdata.org/record/ihme-data/gbd-2019-population-estimates-1950-2019.
4. Wilkinson T, Sculpher MJ, Claxton K, Revill P, Briggs A, Cairns JA, et al. The International Decision Support Initiative Reference Case for Economic Evaluation: An Aid to Thought. Value Health. 2016;19(8):921-8.
5. Ochalek J, Lomas J, Claxton K. Estimating health opportunity costs in low-income and middle-income countries: a novel approach and evidence from cross-country data. BMJ Glob Health. 2018;3(6):e000964.
6. World Development Indicators [Internet]. 2021 [cited 20 June 2021]. Available from: https://databank.worldbank.org/indicator/NY.GDP.PCAP.CD/1ff4a498/Popular-Indicators.
7. Global Burden of Disease Collaborative Network. Global Burden of Disease Study 2019 (GBD 2019) Results Tool (GHDx) Seattle, United States of America: Institute for Health Metrics and Evaluation (IHME); 2020 [updated 29 January 2021. Available from: http://ghdx.healthdata.org/gbd-results-tool.
8. WHO. World malaria report 2020. Geneva, Switzerland: World Health Organization; 2020.
9. U.S. President's Malaria Initiative. Tanzania (Mainland): Malaria Operational Plan FY 2020. Washington, D.C.
10. WHO. Global Health Observatory data repository: Life tables by country Geneva: World Health Organization; 2020 [updated 6 December 2020. Available from: http://apps.who.int/gho/data/view.main.61450?lang=en.
11. Salomon JA, Haagsma JA, Davis A, de Noordhout CM, Polinder S, Havelaar AH, et al. Disability weights for the Global Burden of Disease 2013 study. Lancet Glob Health. 2015;3(11):e712-23.
12. Global Fund. Pooled Procurement Mechanism Reference Pricing: LLINs Geneva: Global Fund; 2021 [updated 15 May 2021. Available from: https://www.theglobalfund.org/media/5861/psm_llinreferenceprices_table_en.pdf?u=637066531800000000.
13. Ministry of Health CD, Gender, Elderly, Children - MoHCDGEC/Tanzania Mainland, Ministry of Health - MoH/Zanzibar, National Bureau of Statistics - NBS/Tanzania, Office of Chief Government Statistician - OCGS/Zanzibar, ICF. Tanzania Demographic and Health Survey and Malaria Indicator Survey 2015-2016. Dar es Salaam, Tanzania: MoHCDGEC, MoH, NBS, OCGS, and ICF; 2016.
14. Global Fund. Pooled Procurement Mechanism Reference Pricing: Malaria Rapid Diagnostic Tests (MRDT) Geneva: Global Fund; 2020 [updated 22 July 2020. Available from: https://www.theglobalfund.org/media/7564/psm_hivrdtreferencepricing_table_en.pdf.
15. Global Fund. Pooled Procurement Mechanism Reference Pricing: Antimalarial medicines Geneva: Global Fund; 2021 [updated Q1 2021. Available from: https://www.theglobalfund.org/media/5812/ppm_actreferencepricing_table_en.pdf?u=637066531880000000.
16. WHO-CHOICE unit cost estimates for service delivery [Internet]. World Health Organization. 2011. Available from: http://www.who.int/choice/country/country_specific/en/.
17. WHO. Global Health Observatory data repository: Life tables by country Geneva: World Health Organization; 2019 [Available from: http://apps.who.int/gho/data/view.main.61450?lang=en.
